# Comparative Transcriptomic Analysis of Spermatozoa From High- and Low-Fertile Crossbred Bulls: Implications for Fertility Prediction

**DOI:** 10.3389/fcell.2021.647717

**Published:** 2021-05-10

**Authors:** Mani Arul Prakash, Arumugam Kumaresan, John Peter Ebenezer Samuel King, Pradeep Nag, Ankur Sharma, Manish Kumar Sinha, Elango Kamaraj, Tirtha Kumar Datta

**Affiliations:** ^1^Theriogenology Laboratory, Veterinary Gynaecology and Obstetrics, Southern Regional Station of Indian Council of Agricultural Research (ICAR)-National Dairy Research Institute, Bengaluru, India; ^2^Animal Genomics Laboratory, Indian Council of Agricultural Research (ICAR), National Dairy Research Institute, Karnal, India

**Keywords:** RNA-seq, crossbred bull, spermatozoa, fertility, biomarker, oxidative phosphorylation

## Abstract

Crossbred bulls produced by crossing *Bos taurus* and *Bos indicus* suffer with high incidence of infertility/subfertility problems; however, the etiology remains poorly understood. The uncertain predictability and the inability of semen evaluation techniques to maintain constant correlation with fertility demand for alternate methods for bull fertility prediction. Therefore, in this study, the global differential gene expression between high- and low-fertile crossbred bull sperm was assessed using a high-throughput RNA sequencing technique with the aim to identify transcripts associated with crossbred bull fertility. Crossbred bull sperm contained transcripts for 13,563 genes, in which 2,093 were unique to high-fertile and 5,454 were unique to low-fertile bulls. After normalization of data, a total of 776 transcripts were detected, in which 84 and 168 transcripts were unique to high-fertile and low-fertile bulls, respectively. A total of 176 transcripts were upregulated (fold change > 1) and 209 were downregulated (<1) in low-fertile bulls. Gene ontology analysis identified that the sperm transcripts involved in the oxidative phosphorylation pathway and biological process such as multicellular organism development, spermatogenesis, and *in utero* embryonic development were downregulated in low-fertile crossbred bull sperm. Sperm transcripts upregulated and unique to low-fertile bulls were majorly involved in translation (biological process) and ribosomal pathway. With the use of RT-qPCR, selected sperm transcripts (*n* = 12) were validated in crossbred bulls (*n* = 12) with different fertility ratings and found that the transcriptional abundance of *ZNF706*, *CRISP2*, *TNP2*, and *TNP1* genes was significantly (*p* < 0.05) lower in low-fertile bulls than high-fertile bulls and was positively (*p* < 0.05) correlated with conception rate. It is inferred that impaired oxidative phosphorylation could be the predominant reason for low fertility in crossbred bulls and that transcriptional abundance of *ZNF706*, *CRISP2*, *TNP2*, and *TNP1* genes could serve as potential biomarkers for fertility in crossbred bulls.

## Introduction

Male fertility is of great importance in dairy cattle breeding industry because semen from a single bull is utilized to breed several thousands of females. The most accurate method for testing the bull fertility is insemination of many fertile females, but this method is time-consuming and expensive, and only a lesser number of males can be tested at any given time (Gillan et al., 2008; Kumaresan et al., 2017). Therefore, it is obligatory to depend on semen fertility prediction techniques (Rodriguez-Martinez, 2003). Available semen evaluation techniques and their foretelling ability of bull fertility are highly variable (Rodríguez-Martínez, 2006; Rodriguez-Martinez and Barth, 2007). Existing semen evaluation assays estimate only few structural attributes of spermatozoa rather than their functional attributes that are having considerable correlation with fertility (Graham, 2001; Kumaresan et al., 2017), which limits the accuracy of bull fertility prediction using these assays. Sperm should comprehend and express many vital attributes in a temporal manner to successfully fertilize the oocyte (Gillan et al., 2008). Bull semen consists of heterogeneous cohorts of subpopulations, as a result of different spermatogenic waves (Rodríguez-Martínez, 2006), and therefore, all the spermatozoa in a given ejaculate are not uniform in terms of their fertilizing capacity. Therefore, it is essential to understand the sperm molecular differences between high- and low-fertile males so that fertility signature molecules could be identified for development of bull fertility prediction tools.

Increasing evidences indicate that males produced by crossing *Bos taurus* males with *Bos indicus* females suffer with high rates of infertility/subfertility; however, the etiology remains poorly understood. Reportedly, a greater proportion of crossbred bulls were culled due to infertility/subfertility and poor semen quality; the average ejaculate rejection rate was around 55% in crossbred bulls (Tyagi et al., 2000; Mukhopadhyay et al., 2010; Vijetha et al., 2014a, b). Therefore, our team has been working toward the aim of decoding the reason behind infertility/subfertility in crossbred bulls, and we found the differences in the proportion of Sertoli cells (Tripathi et al., 2015), metabolomic profile of spermatozoa (Saraf et al., 2020), proteomic profile of seminal plasma (Aslam et al., 2014), spermatozoa (Aslam et al., 2015), spermatogenic and Sertoli cells (Tripathi et al., 2014), and transcriptomic details of testicular tissue (Elango et al., 2020) between crossbred and purebred cattle. However, transcriptomics of crossbred bull sperm is underexplored, although recent studies revealed the relationship of sperm transcripts with sperm function and fertility of purebred bulls (Card et al., 2017; Ren et al., 2017; Burl et al., 2018; Dhawan et al., 2018). Very recently, we reported the transcriptomic profile of crossbred bull spermatozoa that suggested possible implications of transcriptomic variations on semen quality and fertility (Prakash et al., 2020).

Spermatozoa contain both mature and immature RNAs that are represented as a series of exonic, intronic, and intergenic sequences when mapped back to the genome (Selvaraju et al., 2018). A wide variety of coding and non-coding RNA molecules are having important roles in zygote development (Lalancette et al., 2008; Das et al., 2013), post-fertilization events (Selvaraju et al., 2018), early embryonic development (Sendler et al., 2013), epigenetic trans generation inherence (Jodar et al., 2013; Bohacek and Mansuy, 2015; Rando, 2016), and placental development (Selvaraju et al., 2018). Although earlier studies reported that the sire conception rates (CRs) were associated with various levels of mRNAs in Holstein bulls (Card et al., 2017; Dhawan et al., 2018), significant variations in the sperm mRNA transcripts between different breeds (Selvaraju et al., 2017) limit the possibility of having a universal sperm transcript panel for fertility prediction across the breeds, which highlight the need for identification of breed-specific fertility-associated transcripts. Nevertheless, information on fertility-associated sperm transcripts is lacking in crossbred bulls. With this background, in this study, a comprehensive analysis of global differential gene expression between spermatozoa from high- and low-fertile crossbred bulls was carried out using high-throughput RNA sequencing technique with the aim to identify the most relevant molecules for fertility prediction and to understand the reason/pathway behind crossbred bull infertility.

## Materials and Methods

The present study was conducted at Theriogenology Laboratory, ICAR-National Dairy Research Institute, Karnal and Southern Regional Station of ICAR-NDRI, Adugodi, Bengaluru. The study protocol was duly approved by the Institute Animal Ethics Committee (CPCSEA/IAEC/LA/SRS-ICAR-NDRI-2019/No. 09) and performed in accordance with relevant guidelines and regulations.

### Sperm Sample

Cryopreserved semen straws of Holstein Friesian crossbred bulls (*n* = 12) of known fertility status were procured from Kerala Livestock Development Board, Mattupetty, Kerala, India. The CRs of the experimental bulls are shown in the Supplementary Figure 1. CR was calculated based on the number of animals conceived out of total number of animals inseminated (up to three inseminations). The effect of non-genetic factors on CR was studied using least squares analysis of variance for unequal and non-orthogonal data as described previously by Singh et al. (2016). The adjusted CR was used for the calculation of bull fertility. Bulls with CR Mean + 1 standard deviation were considered as high fertile and Mean - 1 standard deviation was considered as low fertile. Spermatozoa from high- (HF) and low-fertile (LF) bulls (*n* = 4 samples) were individually subjected to high-throughput RNA-seq analysis as detailed below.

### Spermatozoa RNA Isolation and cDNA Synthesis

For RNA isolation, pure sperm fraction obtained using 90–45% discontinuous Percoll gradient was used. This procedure eliminated contaminating substances like epithelial cells and semen extender. Total RNA was isolated from frozen sperm using TRIzol (Ambion, Thermo Fisher Scientific, United States) as described by Parthipan et al. (2015) with minor modifications. RNA quantification was done using NanoDrop (ND-1000, Thermo Fisher Scientific, United States). RNA samples with 260/280 ratio of 1.7–2.0 were selected for cDNA synthesis (reverse transcription), which was done using a combination of oligo (dT) and random hexamers with an initial concentration of 50–100 ng of total RNA from each crossbred bull spermatozoa sample using the RevertAid First Strand cDNA Synthesis Kit (Thermo Fisher Scientific, United States, Catalog number K1622) based on the manufacturer’s instructions of 20 μl final volume. The cDNA samples were stored at −20°C until further processing.

### Transcriptome Library Preparation

Total RNA (1 μg) was used to enrich mRNA using NEB Magnetic mRNA Isolation Kit (Illumina, United States). The transcriptome library was prepared using NEB ultra II RNA library prep kit (Illumina, United States) and sequenced using Illumina NextSeq 500 (Illumina, United States) paired-end technology. The enriched mRNA was fragmented (approximately 200 bp) using fragmentation buffer. Random hexamer primers were then added and hybridized to complementary RNA sequences. These short fragments were used as templates to synthesize the first strand of cDNA using reverse transcriptase and dNTP. The DNA–RNA hybrids produced during first strand cDNA synthesis are converted into full-length double-stranded cDNAs using RNase H and *Escherichia coli* DNA polymerase I. The second strand of cDNA was synthesized using second strand enzyme mix and buffer. The double-stranded cDNA fragments were purified using 1.8× AMPure beads. The purified double-stranded cDNA was end repaired to ensure that each molecule was free of overhangs and has 5′ phosphates and 3′ hydroxyls before the adaptor ligation. The adaptor ligated DNA was then purified using AMPure beads and enriched with specific primers, compatible for sequencing on to the Illumina platforms. The final enriched library was purified and quantified by Qubit^®^ Fluorometer, and the size was analyzed by a bio-analyzer.

### Sperm RNA Sequencing and Data Analysis

The cDNA of high-fertile (*n* = 2) and low-fertile (*n* = 2) bulls were sequenced using Illumina NextSeq 500 sequencing system (Sandor^®^ Lifesciences Pvt. Ltd. Banjara Hills, Hyderabad, India) to generate paired-end 76 bp reads. The sequence analysis was done using the online server tool *Galaxy*^1^ (Shannon et al., 2003). Raw data were generated from the four samples, read quality was checked using *FastQC* (Galaxy version 0.72) program, and the reads were then processed with *Cutadapt* tool (Galaxy Version 1.18) (Martin, 2011). Processing includes removal of adapter (AGATCGGAAGA) sequence, length trimming (>15 bp) and quality trimming (30 phred score). With the use of *HISAT2* (Galaxy Version 2.1.0+galaxy4) (Kim et al., 2015), all the four sample processed reads were aligned to the bovine genome (*Bos taurus* UMD 3.1.94/Btau8), and the samples were sorted with aligned sequences using *Samtools* (Galaxy Version 2.0.2) (Li, 2011). The mapped and properly paired sequence to the reference genome was calculated based on tabular descriptive statistics dataset tool *Flagstat* (Galaxy Version 2.0.1) (Li, 2011). With the use of the tool *Cufflink* (Galaxy Version 2.2.1.2) (Trapnell et al., 2010), the presence of individual transcripts and their expression levels were expressed as fragments per kilobase of exon per million fragments mapped (FPKM). The data of cufflink assemblies were then merged using *Cuffmerge* (Galaxy Version 2.2.1.2). Between high- and low-fertile groups, significant changes in transcript expression, splicing, and promoter were studied using *Cuffdiff* (Galaxy Version 2.2.1.5).

The transcripts expressed between the high- and low-fertile groups were categorized as differentially expressed transcripts based on log2 fold change and FPKM value. Transcripts classified based on log2 fold change > +1 (upregulated in low fertile), >−1 (downregulated in low fertile), and between >±1 and < ± 1 (neutral). The transcripts with FPKM present only in high-fertile group were considered as unique to high-fertile bulls, while transcripts with FPKM present only in low-fertile group were classified as unique to low-fertile bulls. All the raw data were uploaded and available in the National Center for Biotechnology Information (NCBI) Sequence Read Archive (SRA) database under PRJNA516089^2^. The total number of sperm transcripts expressed between high- and low-fertile populations was plotted using online tool *Venny* (Version 2.1.0). Selected differentially expressed spermatozoa transcripts were generated as heat map using *Clustvis* (r programming) (Metsalu and Vilo, 2015).

### Gene Ontology and Functional Pathway Analysis

Gene ontology (GO) classification of sperm transcripts was done using *Uniport*^3^ and the Database for Annotation, Visualization, and Integrated Discovery (DAVID) Bioinformatics Resources (v6.8)^4^ and categorized as molecular function (MF), biological process (BP), cellular component (CC), and Kyoto Encyclopedia of Genes and Genomes (KEGG) pathway. The top 10 BPs, CCs, and MFs were plotted as a donut pie chart using *Highcharts*^5^. Pathway enrichment was carried out using Custer Profiler and enriched KEGG functions. Interaction of genes and detailed network analysis of combined GO categories and pathway analysis was performed using *ClueGo* (Version 2.5.4) and *Cluepedia* (Version 1.5.4) plugins in the open source *Cytoscape* (Version 3.7.1)^6^ platform (Locatelli et al., 2019). A network of interactions between related genes were obtained in the form of different layouts. Every analysis was performed with *B. taurus* as background.

### Real-Time Gene Expression Analysis

Twelve genes (*TPT1*, *PFN1*, *ZNF706*, *CRISP2*, *MDB4*, *TNP2*, *ADIPOR1*, *TNP1*, *IQCF1*, *RACK1*, *TMSB10*, and *TSSK6*) were selected based on fold change, FPKM, and functional relevance to sperm fertilizing potential (Table 1) for validation using RT-qPCR in all the 12 bulls including the four bulls whose spermatozoa were subjected to next-generation sequencing (NGS). Primer designing was done using web-based software PRIMER-3 across an exon–exon junctions in order to eliminate contaminating genomic DNA amplification. The annealing temperatures of primers for the selected genes were optimized using PCR (Prima-96plus, Himedia). The cDNAs prepared from different bull semen samples were subjected to RT-qPCR experiment using Insta Q96 Plus Real Time Machine PCR system (HiMedia, India) in a 20 μl reaction comprising 2 μl of cDNA, 0.5 μl of (10 pmol/μl) forward and reverse primers, and 10 μl of Maxima SYBR Green/ROX qPCR master mix 2×. The thermal cycling conditions consisted of initial denaturation at 95°C for 10 min, followed by 40 cycles of 95°C for 15 s, 60°C for 30 s, and 72°C for 30 s. The primer sequence of selected genes for validation using RT-qPCR is given in Supplementary Table 1. All the reactions were performed in duplicates, and qPCR amplification of selected genes with their desired product sizes was confirmed by 2% agarose gel electrophoresis. Relative gene expression levels were determined using 2^–ΔΔCt^ method (Schmittgen and Livak, 2008), where ΔCt = Ct target − Ct internal reference. The fold change was calculated as the mean expression in low-fertile bulls in comparison with high-fertile bulls. *GAPDH* served as the internal reference gene. Initially, we evaluated the expression stability of 10 commonly used housekeeping genes (Supplementary Table 2) and found that *GAPDH* was the most stable internal control gene (ICG) based on the analysis using Genorm, Normfinder, Delta Ct, and the comprehensive ranking by RefFinder (Supplementary Figure 2). The differences in the relative expression of genes between high- and low-fertile groups were assessed for statistical significance using Mann–Whitney *U*-test (SPSS, 22.0, IBM, United States). The difference was considered as significant when *p* < 0.05.

**TABLE 1:** List of genes selected for real-time expression analysis and their reported functions.

**SI no.**	**Gene**	**Log2 (fold change) in NGS**	**Reported functional roles**	**References**
1.	*TPT1*	8.79	Process of apoptosis, cellular differentiation, and control of sperm functions	Arcuri et al., 2004
2.	*PFN1*	6.74	Regulation of actin filament polymerization and oocyte maturation	Rawe et al., 2006
3.	*RACK1*	3.31	Cell regulation, and motility and translation	Gibson, 2012; Gandin et al., 2013
4.	*ZNF706*	−16.03	Sperm functions and male fertility	Kerns et al., 2018
5.	*MDB4*	−5.81	DNA repair and spermatogenesis	Ruddock-D’Cruz et al., 2008; Terribas et al., 2010
6.	*CRISP2*	−5.19	Spermatogenesis, sperm motility, acrosome reaction, capacitation, and fertilization	Card et al., 2013; Zhou et al., 2015; Brukman et al., 2016; Legare et al., 2017
7.	*TNP2*	−2.74	Acrosome reaction, penetration of zona pellucida, and spermatogenesis	Selvaraju et al., 2018; Yathish et al., 2018
8.	*TNP1*	−2.52	Chromatin remodeling, spermatid development, and spermatogenesis	Meistrich et al., 2003; Yathish et al., 2018
9.	*ADIPOR1*	−2.06	Capacitation and sire fertility	Kasimanickam et al., 2013; Kadivar et al., 2016
10.	*TSSK6*	6.9 (FPKM unique to HF)	Gamete fusion, sperm chromatin condensation, and protein phosphorylation	Sosnik et al., 2009; Selvaraju et al., 2018
11.	*IQCF1*	2.89 (FPKM unique to HF)	Motility, capacitation, and acrosome reaction	Fang et al., 2015
12.	*TMSB10*	12.31 (FPKM unique to LF)	Capacitation and fertilization	Selvaraju et al., 2017

*HF, high fertile; LF, low fertile; NGS, next-generation sequencing.*

## Results

### Sperm Transcriptome Profile of High- and Low-Fertile Bulls

**FIGURE 1:**
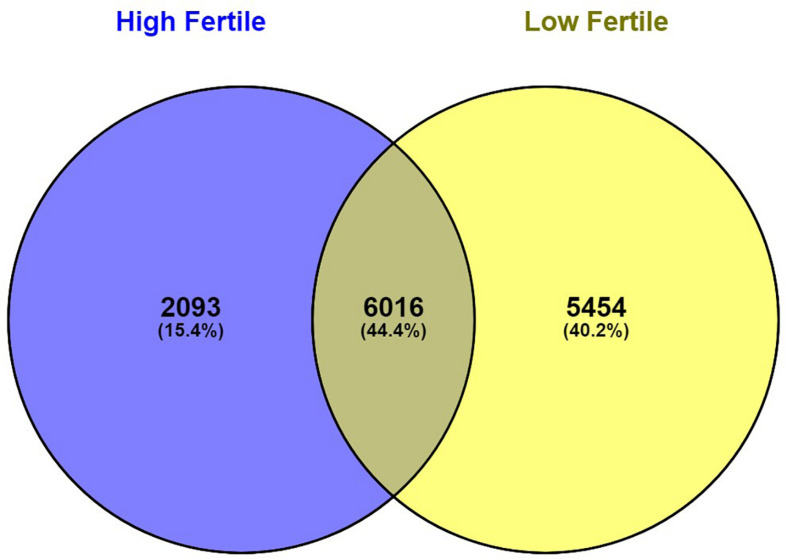
Venn diagram representing total number of observed sperm transcripts identified in high- and low-fertile bull spermatozoa.

**FIGURE 2:**
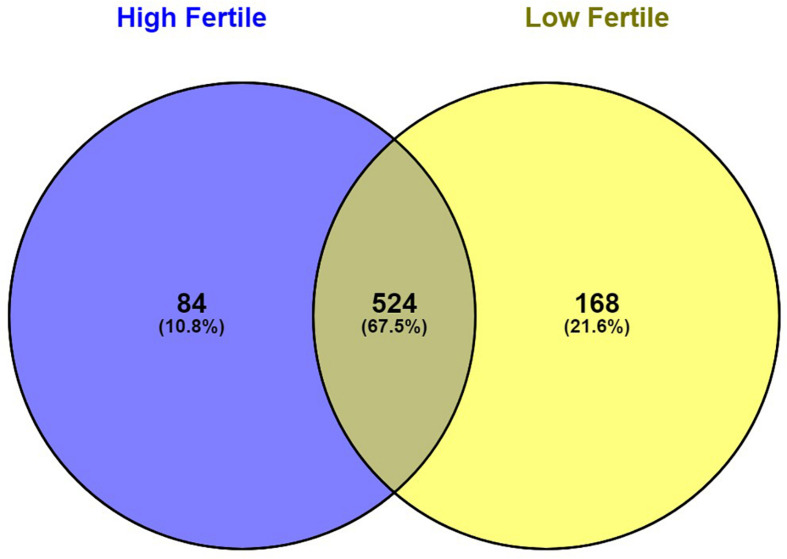
Venn diagram representing the total number of significantly regulated sperm transcripts in high- and low-fertile bull spermatozoa (after normalization).

**FIGURE 3:**
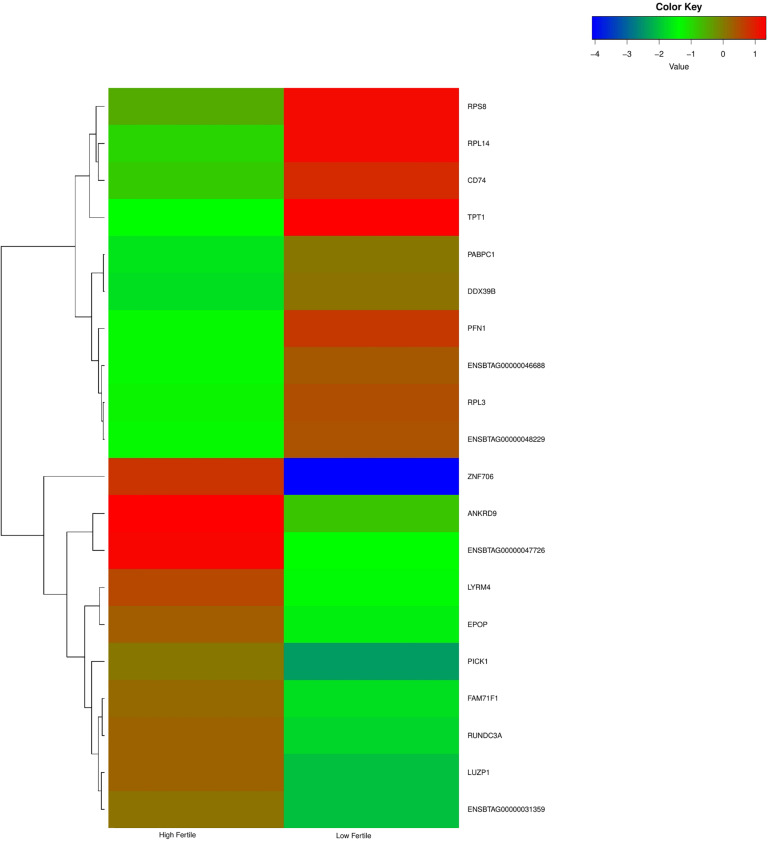
Heat map of top 20 differentially expressed sperm transcripts between high- and low-fertile bulls.

With the use of Illumina Next Seq-500 RNA sequencing, the total raw data generated for the study were 115 million reads. Processing of raw data resulted in 97 million reads, which was mapped against the *Bos taurus* genome. The crossbred bull spermatozoa contained transcripts for 13,563 genes; high- and low-fertile bull spermatozoa contained transcripts for 8,109 and 11,470 genes, respectively. Of the total transcripts detected, 6,016 transcripts were common between the high- and low-fertile populations, 2,093 transcripts were unique to the high-fertile population, and 5,454 transcripts were unique to the low-fertile population (Figure 1). Since there are differences in the total number of reads in each sample and a normalized count is required to compare the samples, we normalized the data using Cufflink tool, which generates normalized read counts in FPKM. By this process, the read count matrix was transformed to allow meaningful comparisons of counts across samples. The existence of specific transcripts and the abundance of mRNA transcripts were represented as FPKM using the Cufflink tool. The processed reads were mapped to the *B. taurus* reference genome. Read mapping to the exonic region was used to measure the relative abundances of mRNA transcripts. Therefore, the normalized expression in FPKM was determined based on the number of reads mapped. After normalization of data, a total of 776 transcripts were detected, in which 524 transcripts were common to both high- and low-fertile bulls, 84 sperm transcripts were unique to high-fertile bulls, and 168 transcripts were unique to low-fertile bulls (Figure 2), while 176 transcripts were upregulated (fold change > 1) and 209 were downregulated (fold change < 1) in low-fertile bulls. The top 20 differentially expressed sperm transcripts were plotted using heat map (Figure 3), in which ribosomal proteins (*RPS8* and *RP14*) and *TPT1* were highly upregulated, whereas *ZNF706* was highly downregulated in low-fertile bulls. The top 10 transcripts unique to the high- and low-fertile populations after excluding the non-coding RNAs are listed in Tables 2, 3.

**TABLE 2:** Top 10 sperm transcripts unique to high-fertile bull spermatozoa.

**SI no.**	**Transcript**	**Description**	**Accession number**	**FPKM**
1.	ENSBTAG00000006046	Protein_coding	N/A	12.76
2.	ENSBTAG00000038366	Protein_coding	N/A	7.58
3.	ENSBTAG00000019880	Protein_coding	N/A	7.07
4.	*TSSK6*	Testis-specific serine kinase 6	XM_002688518	6.90
5.	ENSBTAG00000044154	Protein_coding	N/A	6.07
6.	*C12H13orf46*	Chromosome 12 C13orf46 homolog	NM_001077039	5.25
7.	*FABP3*	Fatty acid-binding protein 3	NM_174313	3.30
8.	ENSBTAG00000047199	Protein_coding	N/A	3.07
9.	*IQCF1*	IQ motif-containing F1	NM_001075773	2.89
10.	ENSBTAG00000030927	Protein_coding	N/A	2.78

*FPKM, fragments per kilobase of exon per million fragments mapped.*

**TABLE 3:** Top 10 sperm transcripts unique to low-fertile bull spermatozoa.

**SI no.**	**Transcript**	**Description**	**Accession number**	**FPKM**
1.	*RPL37*	Ribosomal protein L37	NM_001078132	21.57
2.	*RPS11*	Ribosomal protein S11	NM_001024568	20.97
3.	*RPS12*	Ribosomal protein S12	NM_001014387	17.69
4.	*RPL13A*	Ribosomal protein L13a	NM_001076998	15.35
5.	*RPS3*	Ribosomal protein S3	NM_001034047	13.66
6.	*RPS27*	Ribosomal protein S27	NM_001098135	13.33
7.	*RPL31*	Ribosomal protein L31	NM_001025341	13.30
8.	*TMSB10*	Thymosin beta 10	NM_174623	12.31
9.	*RPL30*	Ribosomal protein L30	NM_001034434	10.56
10.	*RPL32*	Ribosomal protein L32	NM_001034783	8.84

*FPKM, fragments per kilobase of exon per million fragments mapped.*

A complex pool of coding and non-coding RNAs was also observed in the sperm transcriptome. Among the total transcripts (13,563 transcripts), coding RNAs, non-coding RNAs, pseudogenes, and processed pseudogene were 13,002, 88, 375, and 98, respectively. Among the non-coding RNAs, miscellaneous RNA (misc_RNA), small nuclear RNA (snRNA), small nucleolar RNA (snoRNA), and ribosomal RNA (rRNA) were 48, 21, 12, and 7, respectively. After normalization, a total of 776 transcripts were detected, in which coding RNA (known/categorized), coding RNA (unknown/uncategorized), and non-coding RNA were 585, 148, and 43, respectively. Among the non-coding RNAs, misc_RNA, snRNA, snoRNA, and rRNA were 19, 12, 6, and 6, respectively. A total of 61 protein coding ribosomal mRNAs were observed among the total transcripts after normalization.

### Gene Ontology Analysis

**FIGURE 4:**
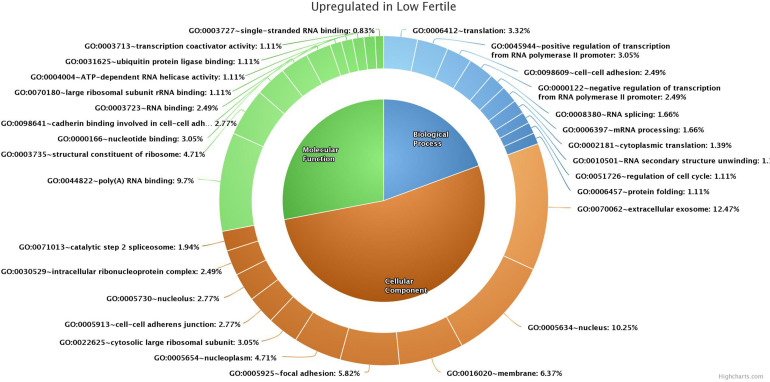
Top 10 gene ontology categories of sperm transcripts upregulated in low-fertile bulls.

**FIGURE 5:**
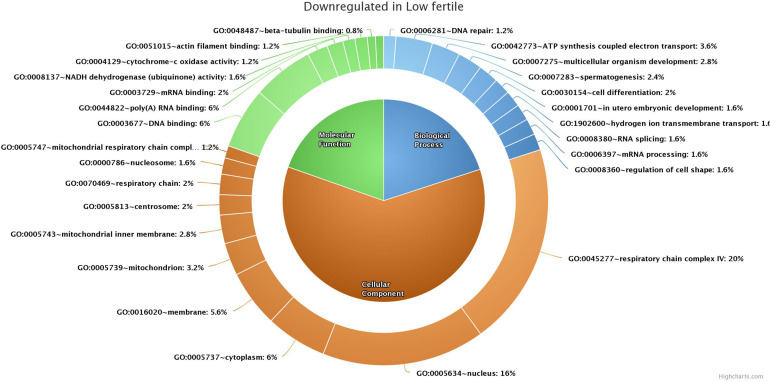
Top 10 gene ontology categories of sperm transcripts downregulated in low-fertile bulls.

GO analysis of 176 (139 functionally annotated) sperm transcripts upregulated in the low-fertile population revealed their involvement in 16 MFs, 39 BPs, and 24 CCs (the top 10 in each GO category are shown in Figure 4) and 12 KEGG pathways (Supplementary Table 3). GO analysis of 209 (178 functionally annotated) sperm transcripts downregulated in the low-fertile population revealed their involvement in 8 MFs, 17 BPs, and 12 CCs (the top 10 in each GO category are shown in Figure 5) and 8 KEGG pathways (Supplementary Table 4). The 524 (436 functionally annotated) sperm transcripts common to the high- and low-fertile populations revealed their involvement in 29 MF, 71 BP, and 39 CC categories and 15 KEGG pathways. The 84 sperm transcripts (50 functionally annotated) unique to the high-fertile population revealed their involvement in 2 MFs and 1 BP as innate immune response with three genes (*PYCARD*, *APCS*, and ENSBTAG00000039963) and 3 KEGG pathways (Supplementary Table 5), while 168 sperm transcripts (118 functionally annotated) unique to the low-fertile population had 10 MFs, 12 BPs, 20 CCs, and 4 KEGG pathways (Supplementary Table 6).

### Pathway and Network Analysis

**FIGURE 6:**
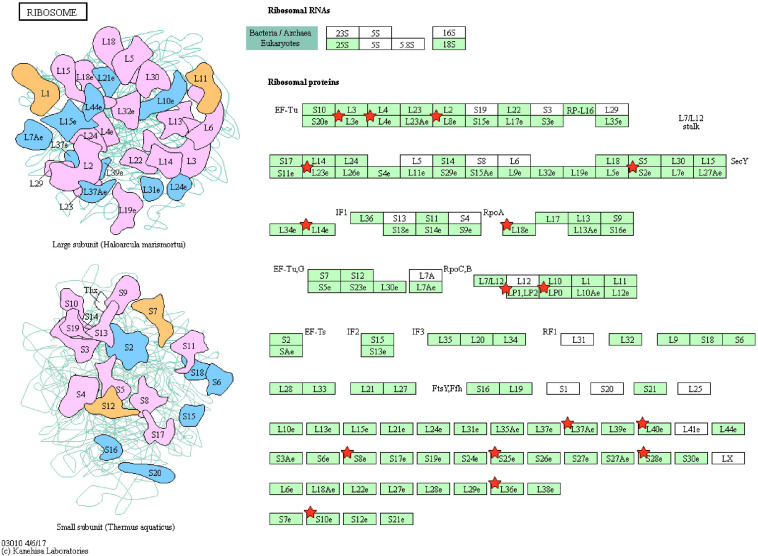
Ribosome pathway with sperm transcripts upregulated in low-fertile bull sperm.

**FIGURE 7:**
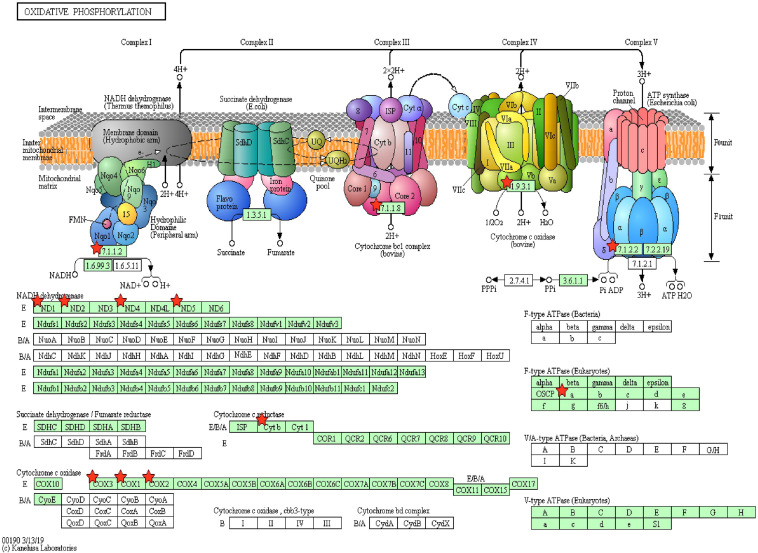
Oxidative phosphorylation pathway with sperm transcripts downregulated in low-fertile bull sperm.

Pathway enrichment of genes specific to sperm transcripts upregulated in the low-fertile populations (12 KEGG pathways) indicated that they are highly involved in the ribosome pathway (Figure 6) (12.23%, 17 counts, 1.09E-12). The transcripts downregulated in the low-fertile population (8 KEGG pathways) were highly involved in oxidative phosphorylation (Figure 7) (5.05%, 9 counts, 7.67E-06). The common transcripts between high and low fertility (15 KEGG pathways) were highly involved in ribosome pathway (4.59%, 20 counts, 2.73E-09). Transcripts unique to high fertility (3 KEGG pathways) were involved in Huntington disease (8%, 4 counts, 0.009), oxidative phosphorylation (6%, 3 counts, 0.038), Parkinson’s disease (6%, 3 counts, 0.044), and transcripts unique to low fertility (4 KEGG pathways) were involved in ribosome (35.59%, 42 counts, 1.86E-51), spliceosome (5.08%, 6 counts, 1.86E-51), antigen processing and presentation (4.23%, 5 counts, 0.009), and phagosome (4.23%, 5 counts, 0.098) pathways.

Network analysis of sperm transcripts upregulated in low fertile expressed 120 BPs (Supplementary Figure 3), 27 CCs, 26 MFs, and 14 KEGG pathways (Supplementary Figure 4). Transcripts downregulated in low-fertile bull spermatozoa revealed 28 BPs (Supplementary Figure 5), 6 CCs, 5 MFs, and 10 KEGG pathways (Supplementary Figure 6). Transcripts unique to high- and low-fertile bull spermatozoa showed 90 BPs, 30 CCs, 10 MFs, and 35 KEGG pathways and 46 BPs, 12 CCs, 4 MFs, and 3 KEGG pathways, respectively.

### Real-Time Expression Analysis of Selected Genes

**FIGURE 8:**
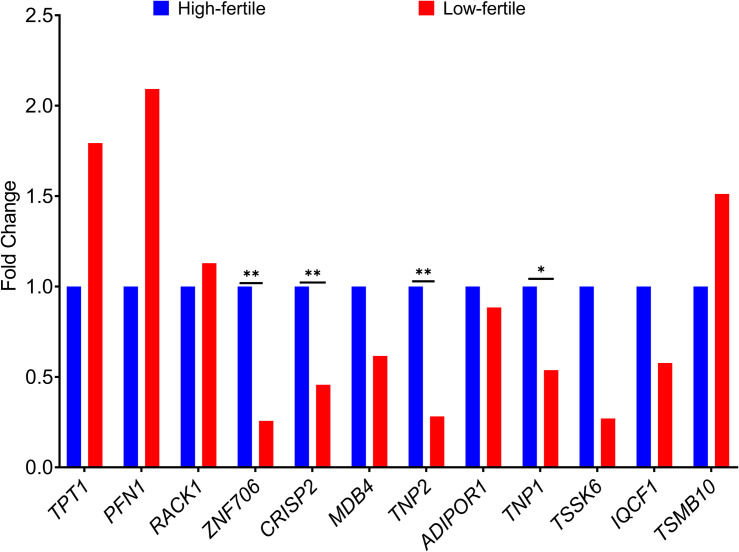
Fold change in expression of selected genes in high- and low-fertile bulls (HF, high-fertile bulls; LF, low-fertile bulls). *p < 0.05; **p < 0.01.

The fold change in the expression of the 12 genes (*TPT1*, *PFN1*, *ZNF706*, *CRISP2*, *MDB4*, *TNP2*, *ADIPOR1*, *TNP1*, *IQCF1*, *RACK1*, *TMSB10*, and *TSSK6*) between high- and low-fertile bulls is given in Figure 8. Results of RT-qPCR expression analysis revealed that all the genes followed the same trend of expression as observed in NGS, except for *RACK1* gene. To understand the variability in the expression of the 12 genes, the geometric mean with 95% confidence interval was calculated for high- and low-fertile bulls, and the results are shown in Supplementary Figure 7. Real-time expression analysis indicated that four genes (*ZNF706*, *CRISP2*, *TNP2*, and *TNP1*) were significantly (*p* < 0.05) downregulated in low-fertile bull spermatozoa. Correlation analysis revealed that expression levels of *ZNF706*, *CRISP2*, *TNP2*, and *TNP1* genes were positively and significantly (*p* < 0.05) related to bull fertility (Table 4).

**TABLE 4:** Relationship among transcriptional abundance of selected genes and bull conception rate.

	***TPT1***	***PFN1***	***ZNF706***	***CRISP2***	***MDB4***	***TNP2***	***ADIPOR1***	***TNP1***	***IQCF1***	***RACK1***	***TMSB10***	***TSSK6***	**CR**
*TPT1*	1	0.944**	−0.412	−0.496	−0.250	−0.427	0.331	−0.168	−0.193	0.738**	0.861**	−0.355	−0.396
*PFN1*		1	−0.379	−0.392	−0.247	−0.307	0.411	−0.095	−0.206	0.801**	0.957**	−0.278	−0.326
*ZNF706*			1	0.803**	0.641*	0.804**	0.518	0.735**	0.576	0.183	−0.252	0.146	0.719**
*CRISP2*				1	0.785**	0.834**	0.424	0.928**	0.602*	0.029	−0.233	0.169	0.820**
*MDB4*					1	0.717**	0.567	0.788**	0.852**	0.118	−0.138	0.051	0.530
*TNP2*						1	0.568	0.802**	0.737**	0.184	−0.137	0.086	0.628*
*ADIPOR1*							1	0.566	0.664*	0.767**	0.429	−0.104	0.177
*TNP1*								1	0.625*	0.311	0.069	0.026	0.770**
*IQCF1*									1	0.249	−0.146	0.023	0.312
*RACK1*										1	0.826**	−0.130	−0.001
*TMSB10*											1	−0.221	−0.176
*TSSK6*												1	0.226
CR													1

CR, conception rate. **Correlation is significant at the 0.01 level (2-tailed). *Correlation is significant at the 0.05 level (2-tailed).

## Discussion

The uncertain predictability and the inability of the currently available semen evaluation techniques to maintain constant correlation with bull fertility made us to look for a sperm transcript-based alternative technique for crossbred bull fertility prediction. In that way, earlier studies on humans and livestock ascertained the role of sperm RNA in spermatogenesis, sperm function, and early embryonic and extra embryonic development (Card et al., 2013; Selvaraju et al., 2018). Therefore, we carried out comparative transcriptomic profiling of high- and low-fertile crossbred bull spermatozoa using high-throughput RNA sequencing technique to decipher the molecular soothsayers for bull fertility and to understand the reason/pathway behind crossbred bull infertility.

The present study revealed the transcripts for 13,814 genes in crossbred bull sperm, which is similar to the earlier studies by and Selvaraju et al. (2017) and Raval et al. (2019), but Card et al. (2013) reported only 6,166 transcripts in bovine sperm. These variations might be due to season of sample collection (Godia et al., 2019), state of spermatozoa (fresh or frozen) (Chen et al., 2015; Card et al., 2017; Singh et al., 2019), method of RNA isolation (Parthipan et al., 2015; Fraser et al., 2017), integrity of sperm RNA, RNA sequencing instrument (Selvaraju et al., 2017), and library preparation methods (Mao et al., 2014). A high proportion of protein coding ribosomal RNAs was detected as unique and upregulated in low-fertile sperm than high-fertile sperm. Ribosomal RNAs were reportedly packed during the process of spermatogenesis (Garrido et al., 2009; Montjean et al., 2012) and essential for the protein synthesis and sperm motility (Bansal et al., 2015), but their implication in crossbred infertility needs to be studied further.

The highly upregulated sperm transcripts in low-fertile crossbred bulls were *TPT1*, *RPL14*, *PFN1*, *DDX39B*, *RPL3*, *PABPC1*, *RPS8*, and *CD74*. The tumor protein, translationally controlled 1 (*TPT1*), has a role in apoptosis, cellular differentiation, and sperm functions (Arcuri et al., 2004). It is reportedly abundant in the sperm of humans (Zhao et al., 2006) and chickens (Singh et al., 2015) but downregulated in oligozoospermic men (Montjean et al., 2012). Ribosomal protein L14 (*RPL14*) is abundant in the testis of *Bos taurus* and *Bos indicus* bulls (Selvaraju et al., 2018; Raval et al., 2019), whereas ribosomal protein S8 (*RPS8*) is abundant in human sperm (Zhao et al., 2006). Profilin 1 (*PFN1*) modulates actin and is involved in oocyte maturation, fertilization, embryo development (Rawe et al., 2006), and spermatogenesis (Selvaraju et al., 2018). DExD-box helicase 39B (*DDX39B*) and poly(A) binding protein cytoplasmic 1 (*PABPC1*) are involved in RNA metabolism (Ozturk et al., 2012; Xu et al., 2014; Raj et al., 2019). *CD74* molecule (*CD74*) controls the antigen presentation for immune responses and reported in the testes of chickens (Singh et al., 2015) and cyprinid fish (Hu et al., 2017).

The highly downregulated sperm transcripts in low-fertile crossbred bull were *ZNF706*, *PICK1*, *LUZP1*, *ANKRD9*, *RUNDC3A*, *LYRM4*, *FAM71F1*, and *EPOP*. The zinc finger protein (*ZNF706*) may be related to zinc that is essential for male fertility and to zinc-containing metalloenzymes that are associated with sperm functions (Kerns et al., 2018). Protein interacting with *PRKCA 1* (*PICK1*) is necessary for cytoskeletal organization (Selvaraju et al., 2017) and acrosome formation (Chen et al., 2016), and its deletion leads to globozoospermia (Liu et al., 2010). Its downregulation in our study is in accordance with Singh et al. (2019). Ankyrin repeat domain 9 (*ANKRD9*) is involved in lipid metabolism (Wang et al., 2009) and downregulated in asthenozoospermic humans (Jodar et al., 2011). RUN domain-containing 3A (*RUNDC34*) and LYR motif-containing 4 (*LYRM4*) are involved in protein biosynthesis. A family with sequence similarity 71 member F1 (*FAM71F1*) was detected in Leydig cells and downregulated in azoospermic males (Malcher et al., 2013). Elongin BC and polycomb repressive complex 2-associated protein (*EPOP*) is a novel gene that has not been reported in bull spermatozoa.

In NGS analysis, we observed that the top sperm transcripts unique to high-fertile bulls were *TSSK6*, *C12H13orf46*, *FABP3*, and *IQCF1*. However, in RT-qPCR analysis, we found that *TSSK6* and *IQCF1* were expressed in both high- and low-fertile bull spermatozoa, but the level of expression was lower in low-fertile bulls. With the available knowledge, although it is difficult to explain this paradoxical situation, in NGS data processing, it may be possible that reads of a particular transcript might be discarded during the process of removal of low-quality reads. Testis-specific serine kinase 6 (*TSSK6*) is involved in protein phosphorylation, sperm chromatin condensation (Selvaraju et al., 2018), sperm motility (Bissonnette et al., 2009; Mondal et al., 2013), and gamete fusion (Sosnik et al., 2009). The function of chromosome 12 C13orf46 homolog (*C12H13orf46*) is unknown. Fatty acid-binding protein 3 (*FABP3*) plays a role in remodeling of member polar lipids in spermatogenesis (Furuhashi and Hotamisligil, 2008), but in contrast to our results, it was highly upregulated in poor motile crossbred semen (Yathish et al., 2017). IQ motif-containing F1 (*IQCF1*) is localized in the acrosome and involved in tyrosine phosphorylation, motility, capacitation, and acrosome reaction (Fang et al., 2015). The top sperm transcripts unique to low-fertile bulls include ribosomal proteins (*RPL37*, *RPS11*, *RPS12*, *RPL13A*, *RPS3*, *RPS27*, *RPL31*, *RPL30*, and *RPL32*) and thymosin beta 10 (*TMSB10*). *TMSB10* is abundant in the embryos of high-fertile sires (Kropp et al., 2017) and is possibly involved in sperm capacitation, fertilization (Selvaraju et al., 2017), and cellular remodeling during trophoblast adhesion (Cammas et al., 2005). Sperm transcripts upregulated and unique to low-fertile bulls were involved in a translation as a BP similar to the reports by Card et al. (2017), Selvaraju et al. (2017), and Raval et al. (2019), and ribosomal pathway. This along with the above-mentioned upregulation and abundance of ribosomal proteins in low-fertile crossbred bulls is collectively indicating the possible changes in the translation machinery in low-fertile crossbred bulls. Previous studies described the importance of ribosomal RNAs for the sperm function (Platts et al., 2007; Bansal et al., 2015); however, the exact role of ribosomal RNAs in male fertility is not yet understood.

The important finding of this study is that the sperm transcripts involved in the multicellular organism development (*QKI*, *ODF1*, *TNP1*, *PRM2*, *CFDP1*, *TNP2*, *ODF2*, *SPEM1*, and *MEA1*), spermatogenesis (*ODF1*, *BCL2L11*, *PRM2*, *TNP2*, *ODF2*, *SPEM1*, and *MEA1*), cell differentiation (*QKI*, *ODF1*, *TNP2*, *ODF2*, *SPEM1*, and *MEA1*), *in utero* embryonic development (*YBX1*, *UBE2B*, *BCL2L11*, *MYH10*, and *RBBP6*), and oxidative phosphorylation pathway (*MT-ATP6*, *ND1*, *MT-ND2*, *MT-ND4*, *ND5*, *MT-CYB*, *COX1*, *MT-CO2*, and *COX3*), which are extremely vital, are downregulated in low-fertile crossbred bull sperm. Oxidative phosphorylation is essential for the sperm to synthesize ATP and produce energy in all mammals (Garrett et al., 2008; Storey, 2008). Alterations in the oxidative phosphorylation process lead to altered sperm function (Terrell et al., 2011) and asthenozoospermia (Rawe et al., 2006; Pelliccione et al., 2011). Therefore, the impaired oxidative phosphorylation could be the predominant contributing factor for crossbred bull infertility.

As per the results of both qPCR and NGS, sperm transcriptional abundance of *ZNF706*, *CRISP2*, *TNP2*, and *TNP1* genes was lower in low-fertile bulls than high-fertile bulls. The relationship of expression levels of these genes with bull fertility was also strong and positive. The zinc finger protein *ZNF706* may be related to zinc that is essential for male fertility and to zinc-containing metalloenzymes that are associated with sperm functions (Kerns et al., 2018). Cysteine-rich secretory protein 2 (*CRISP2*), located in the acrosome and sperm tail (Busso et al., 2005; Jamsai et al., 2010), has a role in spermatogenesis, modulation of flagellar motility, capacitation, acrosome reaction, gamete fusion, and fertilization (Zhao et al., 2006; Arangasamy et al., 2011; Kasimanickam, 2011; Card et al., 2013; Zhou et al., 2015; Brukman et al., 2016; Legare et al., 2017). Reduced *CRISP2* protein levels were found in azoospermia (Du et al., 2006) and asthenozoospermia (Jing et al., 2011; Heidary et al., 2019). Transition nuclear proteins (*TNP1* and *TNP2*) have a role in motility, chromatin integrity, and nucleo-histone to nucleo-protamine transition (Meistrich et al., 2003; Oliva, 2006; Selvaraju et al., 2018; Yathish et al., 2018). The lack of *TNP1* and *TNP2* leads to defects in sperm head, reduced sperm motility, and infertility (Adham et al., 2001; Shirley et al., 2004).

## Conclusion

In conclusion, our study established the transcriptomic profile on high- and low-fertile crossbred bull spermatozoa using high-throughput RNA sequencing. These RNAs might have a titanic role on spermatogenesis, post-spermatogenic events, sperm functions, fertilization, and early embryonic development. We identified that spermatozoa from low-fertile bulls had an altered expression of genes involved in oxidative phosphorylation, sperm functions, and embryonic development. The impaired function of oxidative phosphorylation could be the predominant reason for crossbred bull infertility; and significant downregulation of *ZNF706*, *CRISP2*, *TNP2*, and *TNP1* genes indicates that they could serve as potential biomarkers for fertility in crossbred bulls.

## Data Availability Statement

The datasets presented in this study can be found in online repositories. The names of the repository/repositories and accession number(s) can be found below: https://www.ncbi.nlm.nih.gov/, PRJNA516089.

## Ethics Statement

The animal study was reviewed and approved by Institute Animal Ethics Committee, SRS of ICAR-NDRI.

## Author Contributions

MAP: methodology, experiment, writing – original draft, and data curation. AK: conceptualization, project administration, supervision, writing – review, and editing. JE, PN, and AS: methodology and data curation. MS: data curation and bioinformatic analysis. EK: methodology and writing – original draft. TD: formal analysis, writing – review, and editing. All authors contributed to the article and approved the submitted version.

## Conflict of Interest

The authors declare that the research was conducted in the absence of any commercial or financial relationships that could be construed as a potential conflict of interest.

## Footnotes

1 https://usegalaxy.org/

2 https://www.ncbi.nlm.nih.gov/sra/PRJNA516089

3 https://www.uniprot.org/

4 https://david.ncifcrf.gov/

5 https://www.highcharts.com/demo/pie-donut

6 https://Cluego.org/

## References

[B1] Adham I. M. Nayernia K. Burkhardt-Göttges E. Topaloglu Ö Dixkens C. Holstein A. F. (2001). Teratozoospermia in mice lacking the transition protein 2 (Tnp2). *Mol. Hum. Reprod.* 7 513–520. 10.1093/molehr/7.6.513 11385107

[B2] Arangasamy A. Kasimanickam V. R. DeJarnette J. M. Kasimanickam R. K. (2011). Association of CRISP2, CCT8, PEBP1 mRNA abundance in sperm and sire conception rate in Holstein bulls. *Theriogenology* 76 570–577. 10.1016/j.theriogenology.2011.03.009 21529916

[B3] Arcuri F. Papa S. Carducci A. Romagnoli R. Liberatori S. Riparbelli M. G. (2004). Translationally controlled tumor protein (TCTP) in the human prostate and prostate cancer cells: expression, distribution, and calcium binding activity. *Prostate* 60 130–140. 10.1002/pros.20054 15162379

[B4] Aslam M. K. M. Kumaresan A. Rajak S. K. Tajmul M. Datta T. K. Mohanty T. K. (2015). Comparative proteomic analysis of Taurine, Indicine, and crossbred (*Bos taurus* × Bos indicus) bull spermatozoa for identification of proteins related to sperm malfunctions and subfertility in crossbred bulls. *Theriogenology* 84 624–633. 10.1016/j.theriogenology.2015.04.020 26033646

[B5] Aslam M. K. M. Kumaresan A. Sharma V. K. Tajmul M. Chhillar S. Chakravarty A. K. (2014). Identification of putative fertility markers in seminal plasma of crossbred bulls through differential proteomics. *Theriogenology* 82 1254–1262. 10.1016/j.theriogenology.2014.08.007 25258256

[B6] Bansal S. K. Gupta N. Sankhwar S. N. Rajender S. (2015). Differential genes expression between fertile and infertile spermatozoa revealed by transcriptome analysis. *PLoS One* 10:e0127007. 10.1371/journal.pone.0127007 25973848 PMC4431685

[B7] Bissonnette N. Levesque-Sergerie J. P. Thibault C. Boissonneault G. (2009). Spermatozoal transcriptome profiling for bull sperm motility: a potential tool to evaluate semen quality. *Reproduction* 138 65–80. 10.1530/REP-08-0503 19423662

[B8] Bohacek J. Mansuy I. M. (2015). Molecular insights into transgenerational non-genetic inheritance of acquired behaviours. *Nat. Rev. Genet.* 16 641–652. 10.1038/nrg3964 26416311

[B9] Brukman N. G. Miyata H. Torres P. Lombardo D. Caramelo J. J. Ikawa M. (2016). Fertilization defects in sperm from Cysteine-rich secretory protein 2 (Crisp2) knockout mice: implications for fertility disorders. *Mol. Hum. Reprod.* 22 240–251. 10.1093/molehr/gaw005 26786179

[B10] Burl R. B. Clough S. Sendler E. Estill M. Krawetz S. A. (2018). Sperm RNA elements as markers of health. *Syst. Biol. Reprod. Med.* 64 25–38. 10.1080/19396368.2017.1393583 29199464

[B11] Busso D. Cohen D. J. Hayashi M. Kasahara M. Cuasnicu P. S. (2005). Human testicular protein TPX1/CRISP-2: localization in spermatozoa, fate after capacitation and relevance for gamete interaction. *Mol. Hum. Reprod.* 11 299–305. 10.1093/molehr/gah156 15734896

[B12] Cammas L. Reinaud P. Dubois O. Bordas N. Germain G. Charpigny G. (2005). Identification of differentially regulated genes during elongation and early implantation in the ovine trophoblast using complementary DNA array screening. *Biol. Reprod.* 72 960–967. 10.1095/biolreprod.104.034801 15616222

[B13] Card C. J. Anderson E. J. Zamberlan S. Krieger K. E. Kaproth M. Sartini B. L. (2013). Cryopreserved bovine spermatozoal transcript profile as revealed by high-throughput ribonucleic acid sequencing. *Biol. Reprod.* 88:49. 10.1095/biolreprod.112.103788 23303677

[B14] Card C. J. Krieger K. E. Kaproth M. Sartini B. L. (2017). Oligo-dT selected spermatozoal transcript profiles differ among higher and lower fertility dairy sires. *Anim. Reprod. Sci.* 177 105–123. 10.1016/j.anireprosci.2016.12.011 28081858

[B15] Chen S. R. Batool A. Wang Y. Q. Hao X. X. Chang C. S. Cheng C. Y. (2016). The control of male fertility by spermatid-specific factors: searching for contraceptive targets from spermatozoon’s head to tail. *Cell Death Dis.* 7:e2472. 10.1038/cddis.2016.344 27831554 PMC5260884

[B16] Chen X. Wang Y. Zhu H. Hao H. Zhao X. Qin T. (2015). Comparative transcript profiling of gene expression of fresh and frozen–thawed bull sperm. *Theriogenology* 83 504–511. 10.1016/j.theriogenology.2014.10.015 25459024

[B17] Das P. J. McCarthy F. Vishnoi M. Paria N. Gresham C. Li G. (2013). Stallion sperm transcriptome comprises functionally coherent coding and regulatory RNAs as revealed by microarray analysis and RNA-seq. *PLoS One* 8:e56535. 10.1371/journal.pone.0056535 23409192 PMC3569414

[B18] Dhawan V. Kumar M. Dadhwal V. Singh N. Dada R. (2018). Sperm transcripts and genomic integrity: role in implantation and embryo viablity in IVF cycles. *Fertil. Steril.* 110 e93–e94. 10.1016/j.fertnstert.2018.07.281

[B19] Du Y. Huang X. Li J. Hu Y. Zhou Z. Sha J. (2006). Human testis specific protein 1 expression in human spermatogenesis and involvement in the pathogenesis of male infertility. *Fertil. Steril.* 85 1852–1854. 10.1016/j.fertnstert.2005.11.064 16759931

[B20] Elango K. Kumaresan A. Sharma A. Nag P. Prakash M. A. Sinha M. K. (2020). Sub-fertility in crossbred bulls: deciphering testicular level transcriptomic alterations between zebu (Bos indicus) and crossbred (*Bos taurus* x Bos indicus) bulls. *BMC Genomics* 21:502. 10.1186/s12864-020-06907-1 32693775 PMC7372791

[B21] Fang P. Xu W. Li D. Zhao X. Dai J. Wang Z. (2015). A novel acrosomal protein, IQCF1, involved in sperm capacitation and the acrosome reaction. *Andrology* 3 332–344. 10.1111/andr.296 25380116

[B22] Fraser L. Brym P. Pareek C. S. (2017). Isolation of total ribonucleic acid from fresh and frozen-thawed boar semen and its relevance in transcriptome studies. *S. Afr. J. Anim. Sci.* 47 56–60. 10.4314/sajas.v47i1.9

[B23] Furuhashi M. Hotamisligil G. S. (2008). Fatty acid-binding proteins: role in metabolic diseases and potential as drug targets. *Nat. Rev. Drug Discov.* 7:489. 10.1038/nrd2589 18511927 PMC2821027

[B24] Gandin V. Senft D. Topisirovic I. Ronai Z. A. (2013). RACK1, Function in cell motility and protein synthesis. *Genes. Cancer* 4 369–377. 10.1177/1947601913486348 24349634 PMC3863339

[B25] Garrett L. J. Revell S. G. Leese H. J. (2008). Adenosine triphosphate production by bovine spermatozoa and its relationship to semen fertilizing ability. *J. Androl.* 29 449–458. 10.2164/jandrol.107.003533 18046050

[B26] Garrido N. Martinez-Conejero J. A. Jauregui J. Horcajadas J. A. Simon C. Remohi J. (2009). Microarray analysis in sperm from fertile and infertile men without basic sperm analysis abnormalities reveals a significantly different transcriptome. *Fertil. Steril.* 91 1307–1310. 10.1016/j.fertnstert.2008.01.078 18367176

[B27] Gibson T. J. (2012). RACK1 research – ships passing in the night? *FEBS Lett.* 586 2787–2789. 10.1016/j.febslet.2012.04.048 22580388

[B28] Gillan L. Kroetsch T. Maxwell W. C. Evans G. (2008). Assessment of in vitro sperm characteristics in relation to fertility in dairy bulls. *Anim. Reprod. Sci.* 103 201–214. 10.1016/j.anireprosci.2006.12.010 17208395

[B29] Godia M. Estill M. Castello A. Balasch S. Rodríguez-Gil J. E. Sanchez Bonastre A. (2019). A RNA-seq analysis to describe the boar sperm transcriptome and its seasonal changes. *Front. Genet.* 10:299. 10.3389/fgene.2019.00299 31040860 PMC6476908

[B30] Graham J. K. (2001). Assessment of sperm quality: a flow cytometric approach. *Anim. Reprod. Sci.* 68 239–247. 10.1016/s0378-4320(01)00160-9 11744268

[B31] Heidary Z. Zaki-Dizaji M. Saliminejad K. Khorramkhorshid H. R. (2019). Expression Analysis of the CRISP2, CATSPER1, PATE1 and SEMG1 in the Sperm of Men with Idiopathic Asthenozoospermia. *J. Reprod. Infertil.* 20:70. 31058050 PMC6486568

[B32] Hu F. Xu K. Zhou Y. Wu C. Wang S. Xiao J. (2017). Different expression patterns of sperm motility-related genes in testis of diploid and tetraploid cyprinid fish. *Biol. Reprod.* 96 907–920. 10.1093/biolre/iox010 28340181 PMC5441299

[B33] Jamsai D. Rijal S. Bianco D. M. O’Connor A. E. Merriner D. J. Smith S. J. (2010). A novel protein, sperm head and tail associated protein (SHTAP), interacts with cysteine−rich secretory protein 2 (CRISP2) during spermatogenesis in the mouse. *Biol. Cell* 102 93–106. 10.1042/BC20090099 19686095

[B34] Jing X. W. Xing R. W. Zhou Q. Z. Yu Q. F. Guo W. B. Chen S. M. (2011). Expressions of cysteine-rich secretory protein 2 in asthenospermia. *Natl. J. Androl.* 17 203–207. 10.1095/biolreprod.114.124487 21485539

[B35] Jodar M. Oriola J. Mestre G. Castillo J. Giwercman A. Vidal−Taboada J. M. (2011). Polymorphisms, haplotypes and mutations in the protamine 1 and 2 genes. *Int. J. Androl.* 34 470–485. 10.1111/j.1365-2605.2010.01115.x 21029114

[B36] Jodar M. Selvaraju S. Sendler E. Diamond M. P. Krawetz S. A. Reproductive Medicine Network. (2013). The presence, role and clinical use of spermatozoal RNAs. *Hum. Reprod. Update* 19 604–624. 10.1093/humupd/dmt031 23856356 PMC3796946

[B37] Kadivar A. Heidari Khoei H. Hassanpour H. Golestanfar A. Ghanaei H. (2016). Correlation of adiponectin mRNA abundance and its receptors with quantitative parameters of sperm motility in rams. *Int. J. Fertil. Steril.* 10 127–135. 10.22074/ijfs.2016.4778 27123210 PMC4845523

[B38] Kasimanickam R. (2011). “Application of technology in male reproduction,” in *Proceedings of the Applied Reproductive Strategies in Beef Cattle*, Pullman, WA: Department of Veterinary Clinical SciencesWashington State University, 30.

[B39] Kasimanickam V. R. Kasimanickam R. K. Kastelic J. P. Stevenson J. S. (2013). Associations of adiponectin and fertility estimates in Holstein bulls. *Theriogenology* 79 766𠄓77.e1-3. 10.1016/j.theriogenology.2012.12.001 23312718

[B40] Kerns K. Zigo M. Sutovsky P. (2018). Zinc: a necessary ion for mammalian sperm fertilization competency. *Int. J. Mol. Sci.* 19:4097. 10.3390/ijms19124097 30567310 PMC6321397

[B41] Kim D. Langmead B. Salzberg S. L. (2015). HISAT: a fast-spliced aligner with low memory requirements. *Nat. Methods* 12:357. 10.1038/nmeth.3317 25751142 PMC4655817

[B42] Kropp J. Carrillo J. A. Namous H. Daniels A. Salih S. M. Song J. (2017). Male fertility status is associated with DNA methylation signatures in sperm and transcriptomic profiles of bovine preimplantation embryos. *BMC Genomics* 18:280. 10.1186/s12864-017-3673-y 28381255 PMC5382486

[B43] Kumaresan A. Johannisson A. Bergqvist A. S. (2017). Sperm function during incubation with oestrus oviductal fluid differs in bulls with different fertility. *Reprod. Fert. Dev.* 29 1096–1106. 10.1071/RD15474 27112984

[B44] Lalancette C. Thibault C. Bachand I. Caron N. Bissonnette N. (2008). Transcriptome analysis of bull semen with extreme non-return rate: use of suppression-subtractive hybridization to identify functional markers for fertility. *Biol. Reprod.* 78 618–635. 10.1095/biolreprod.106.059030 18003951

[B45] Legare C. Akintayo A. Blondin P. Calvo E. Sullivan R. (2017). Impact of male fertility status on the transcriptome of the bovine epididymis. *Mol. Hum. Reprod.* 23 355–369. 10.1093/molehr/gax019 28379507

[B46] Li H. (2011). Improving SNP discovery by base alignment quality. *Bioinformatics* 27 1157–1158. 10.1093/bioinformatics/btr076 21320865 PMC3072548

[B47] Liu G. Shi Q. W. Lu G. X. (2010). A newly discovered mutation in PICK1 in a human with globozoospermia. *Asian J. Androl.* 12:556. 10.1038/aja.2010.47 20562896 PMC3739375

[B48] Locatelli Y. Forde N. Blum H. Graf A. Piégu B. Mermillod P. (2019). Relative effects of location relative to the corpus luteum and lactation on the transcriptome of the bovine oviduct epithelium. *BMC Genomics* 20:233. 10.1186/s12864-019-5616-2 30898106 PMC6427878

[B49] Malcher A. Rozwadowska N. Stokowy T. Kolanowski T. Jedrzejczak P. Zietkowiak W. (2013). Potential biomarkers of nonobstructive azoospermia identified in microarray gene expression analysis. *Fertil. Steril.* 100 1686–1694. 10.1016/j.fertnstert.2013.07.1999 24012201

[B50] Mao S. Sendler E. Goodrich R. J. Hauser R. Krawetz S. A. (2014). A comparison of sperm RNA-seq methods. *Syst. Biol. Reprod. Med.* 60 308–315. 10.3109/19396368.2014.944318 25077492 PMC4435722

[B51] Martin M. (2011). Cutadapt removes adapter sequences from high-throughput sequencing reads . *EMBnet. J.* 17 10–12. 10.14806/ej.17.1.200

[B52] Meistrich M. L. Mohapatra B. Shirley C. R. Zhao M. (2003). Roles of transition nuclear proteins in spermiogenesis. *Chromosoma* 111 483–488. 10.1007/s00412-002-0227-z 12743712

[B53] Metsalu T. Vilo J. (2015). ClustVis: a web tool for visualizing clustering of multivariate data using principal component analysis and heatmap. *Nucleic Acids Res.* 43 566–570. 10.1093/nar/gkv468 25969447 PMC4489295

[B54] Mondal M. Baruah K. K. Chatterjee A. Ghosh M. K. (2013). Characterization and gene expression profiling of epididymal sperm collected from dead mithun (*Bos Frontalis*) bulls and its preservation. *Int. J. Biotechnol. Bioeng. Res.* 4 535–542.

[B55] Montjean D. De La Grange P. Gentien D. Rapinat A. Belloc S. Cohen-Bacrie P. (2012). Sperm transcriptome profiling in oligozoospermia. *J. Assist. Reprod. Genet.* 29 3–10. 10.1007/s10815-011-9644-3 21989496 PMC3252406

[B56] Mukhopadhyay C. S. Gupta A. K. Yadav B. R. Khate K. Raina V. S. Mohanty T. K. (2010). Subfertility in males: an important cause of bull disposal in bovines. *Asian Australas. J. Anim. Sci.* 23 450–455. 10.5713/ajas.2012.12413 25049791 PMC4093486

[B57] Oliva R. (2006). Protamines and male infertility. *Hum. Reprod.* 12 417–435. 10.1093/humupd/dml009 16581810

[B58] Ozturk S. Guzeloglu-Kayisli O. Demir N. Sozen B. Ilbay O. Lalioti M. D. (2012). Epab and Pabpc1 are differentially expressed during male germ cell development. *Reprod. Sci.* 19 911–922. 10.1177/1933719112446086 22814100 PMC4046314

[B59] Parthipan S. Selvaraju S. Somashekar L. Kolte A. P. Arangasamy A. Ravindra J. P. (2015). Spermatozoa input concentrations and RNA isolation methods on RNA yield and quality in bull (*Bos taurus*). *Anal. Biochem.* 482 32–39. 10.1016/j.ab.2015.03.022 25823682

[B60] Pelliccione F. Micillo A. Cordeschi G. D’Angeli A. Necozione S. Gandini L. (2011). Altered ultrastructure of mitochondrial membranes is strongly associated with unexplained asthenozoospermia. *Fertil. Steril.* 95 641–646. 10.1016/j.fertnstert.2010.07.1086 20840880

[B61] Platts A. E. Dix D. J. Chemes H. E. Thompson K. E. Goodrich R. Rockett J. C. (2007). Success and failure in human spermatogenesis as revealed by teratozoospermic RNAs. *Hum. Mol. Genet.* 16 763–773. 10.1093/hmg/ddm012 17327269

[B62] Prakash M. A. Kumaresan A. Sinha M. K. Kamaraj E. Mohanty T. K. Datta T. K. (2020). RNA-Seq analysis reveals functionally relevant coding and non-coding RNAs in crossbred bull spermatozoa. *Anim. Reprod. Sci.* 222:106621. 10.1016/j.anireprosci.2020.106621 33069132 PMC7607363

[B63] Raj S. Bagchi D. Orero J. V. Banroques J. Tanner N. K. Croquette V. (2019). Mechanistic characterization of the DEAD-box RNA helicase Ded1 from yeast as revealed by a novel technique using single-molecule magnetic tweezers. *Nucleic Acids Res.* 47 3699–3710. 10.1093/nar/gkz057 30993346 PMC6468243

[B64] Rando O. J. (2016). Intergenerational transfer of epigenetic information in sperm. *Cold Spring Harb. Perspect. Med.* 6:a022988. 10.1101/cshperspect.a022988 26801897 PMC4852801

[B65] Raval N. P. Shah T. M. George L. B. Joshi C. G. (2019). Insight into bovine (Bos indicus) spermatozoal whole transcriptome profile. *Theriogenology* 129 8–13. 10.1016/j.theriogenology.2019.01.037 30784792

[B66] Rawe V. Y. Payne C. Schatten G. (2006). Profilin and actin-related proteins regulate microfilament dynamics during early mammalian embryogenesis. *Hum. Reprod.* 21 1143–1153. 10.1093/humrep/dei480 16428331

[B67] Ren X. Chen X. Wang Z. Wang D. (2017). Is transcription in sperm stationary or dynamic? *J. Reprod. Dev.* 63 439–443. 10.1262/jrd.2016-093 28845020 PMC5649092

[B68] Rodriguez-Martinez H. (2003). Laboratory semen assessment and prediction of fertility: still utopia? *Reprod. Domest. Anim.* 38 312–318. 10.1046/j.1439-0531.2003.00436.x 12887570

[B69] Rodríguez-Martínez H. (2006). Can we increase the estimative value of semen assessment? *Reprod. Domest. Anim.* 41 2–10. 10.1111/j.1439-0531.2006.00764.x 16984464

[B70] Rodriguez-Martinez H. Barth A. D. (2007). In vitro evaluation of sperm quality related to in vivo function and fertility. *Soc. Reprod. Fertil. Suppl.* 64 39–54. 10.5661/rdr-vi-39 17494215

[B71] Ruddock-D’Cruz N. T. Xue J. Wilson K. J. Heffernan C. Prashadkumar S. Cooney M. A. (2008). Dynamic changes in the localization of five members of the methyl binding domain (MBD) gene family during murine and bovine preimplantation embryo development. *Mol. Reprod. Dev.* 75 48–59. 10.1002/mrd.20712 17546630

[B72] Saraf K. K. Kumaresan A. Dasgupta M. Karthikkeyan G. Prasad T. S. K. Modi P. K. (2020). Metabolomic fingerprinting of bull spermatozoa for identification of fertility signature metabolites. *Mol. Reprod. Dev.* 87 692–703. 10.1002/mrd.23354 32452071

[B73] Schmittgen T. D. Livak K. J. (2008). Analyzing real-time PCR data by the comparative C T method. *Nat. Protoc.* 3 1101–1108. 10.1038/nprot.2008.73 18546601

[B74] Selvaraju S. Parthipan S. Somashekar L. Binsila B. K. Kolte A. P. Arangasamy A. (2018). Current status of sperm functional genomics and its diagnostic potential of fertility in bovine (*Bos taurus*). *Syst. Biol. Reprod. Med.* 64 484–501. 10.1080/19396368.2018.1444816 29537884

[B75] Selvaraju S. Parthipan S. Somashekar L. Kolte A. P. Binsila B. K. Arangasamy A. (2017). Occurrence and functional significance of the transcriptome in bovine (*Bos taurus*) spermatozoa. *Sci. Rep.* 7:42392. 10.1038/srep42392 28276431 PMC5343582

[B76] Sendler E. Johnson G. D. Mao S. Goodrich R. J. Diamond M. P. Hauser R. (2013). Stability, delivery and functions of human sperm RNAs at fertilization. *Nucleic Acids Res.* 41 4104–4117. 10.1093/nar/gkt132 23471003 PMC3627604

[B77] Shannon P. Markiel A. Ozier O. Baliga N. S. Wang J. T. Ramage D. (2003). Cytoscape: a software environment for integrated models of biomolecular interaction networks. *Genome Res.* 13 2498–2504. 10.1101/gr.1239303 14597658 PMC403769

[B78] Shirley C. R. Hayashi S. Mounsey S. Yanagimachi R. Meistrich M. L. (2004). Abnormalities and reduced reproductive potential of sperm from Tnp1-and Tnp2-null double mutant mice. *Biol. Reprod.* 71 1220–1229. 10.1095/biolreprod.104.029363 15189834

[B79] Singh R. K. Kumaresan A. Chhillar S. Rajak S. K. Tripathi U. K. Nayak S. (2016). Identification of suitable combinations of in vitro sperm-function test for the prediction of fertility in buffalo bull. *Theriogenology* 86 2263–2271. 10.1016/j.theriogenology.2016.07.022 27555524

[B80] Singh R. P. Shafeeque C. M. Sharma S. K. Singh R. Mohan J. Sastry K. V. H. (2015). Chicken sperm transcriptome profiling by microarray analysis. *Genome* 59 185–196. 10.1139/gen-2015-0106 26868024

[B81] Singh R. Junghare V. Hazra S. Singh U. Sengar G. S. Raja T. V. (2019). Database on spermatozoa transcriptogram of catagorised Frieswal crossbred (Holstein Friesian X Sahiwal) bulls. *Theriogenology* 129 130–145. 10.1016/j.theriogenology.2019.01.025 30844654

[B82] Sosnik J. Miranda P. V. Spiridonov N. A. Yoon S. Y. Fissore R. A. Johnson G. R. (2009). Tssk6 is required for Izumo relocalization and gamete fusion in the mouse. *J. Cell Sci.* 122 2741–2749. 10.1242/jcs.047225 19596796 PMC2909320

[B83] Storey B. T. (2008). Mammalian sperm metabolism: oxygen and sugar, friend and foe. *Int. J. Dev. Biol.* 52 427–437. 10.1387/ijdb.072522bs 18649255

[B84] Terrell K. A. Wildt D. E. Anthony N. M. Bavister B. D. Leibo S. P. Penfold L. M. (2011). Oxidative phosphorylation is essential for felid sperm function, but is substantially lower in cheetah (*Acinonyx jubatus*) compared to domestic cat (Felis catus) ejaculate. *Biol. Reprod.* 85 473–481. 10.1095/biolreprod.111.092106 21593479

[B85] Terribas E. Bonache S. García−Arévalo M. Sánchez J. Franco E. Bassas L. (2010). Changes in the expression profile of the meiosis−involved mismatch repair genes in impaired human spermatogenesis. *J. Androl.* 31 346–357. 10.2164/jandrol.109.008805 20075417

[B86] Trapnell C. Williams B. A. Pertea G. Mortazavi A. Kwan G. Van Baren M. J. (2010). Transcript assembly and quantification by RNA-Seq revealsunannotated transcripts and isoform switching during cell differentiation. *Nat. Biotechnol.* 28:511. 10.1038/nbt.1621 20436464 PMC3146043

[B87] Tripathi U. K. Aslam M. K. Pandey S. Nayak S. Chhillar S. Srinivasan A. (2014). Differential proteomic profile of spermatogenic and Sertoli cells from peri-pubertal testes of three different bovine breeds. *Front. Cell Dev. Biol.* 2:24. 10.3389/fcell.2014.00024 25364731 PMC4206989

[B88] Tripathi U. K. Chhillar S. Kumaresan A. Aslam M. M. Rajak S. K. Nayak S. (2015). Morphometric evaluation of seminiferous tubule and proportionate numerical analysis of Sertoli and spermatogenic cells indicate differences between crossbred and purebred bulls. *Vet. World* 8:645. 10.14202/vetworld.2015.645-650 27047150 PMC4774728

[B89] Tyagi S. Mathur A. K. Agarwal S. C. (2000). Semen production performance of Frieswal bulls. *Indian J. Anim. Sci.* 70 1032–1034.

[B90] Vijetha B. T. Layek S. S. Kumaresan A. Mohanty T. K. Gupta A. K. Chakravarty A. K. (2014a). Effects of pedigree and exotic genetic inheritance on semen production traits of dairy bulls. *Asian Pac. J. Reprod.* 3 13–17. 10.1016/S2305-0500(13)60178-5

[B91] Vijetha B. T. Rajak S. K. Layek S. S. Kumaresan A. Mohanty T. K. Chakravarty A. K. (2014b). Breeding soundness evaluation in crossbred bulls: can testicular measurements be used as a tool to predict ejaculate quality. *Indian J. Anim. Sci.* 84 177–180.

[B92] Wang Z. Gerstein M. Snyder M. (2009). RNA-Seq: a revolutionary tool for transcriptomics. *Nat. Rev. Genet.* 10:57. 10.1038/nrg2484 19015660 PMC2949280

[B93] Xu K. Yang L. Zhao D. Wu Y. Qi H. (2014). AKAP3 synthesis is mediated by RNA binding proteins and PKA signaling during mouse spermiogenesis. *Biol. Reprod.* 90:119. 10.1095/biolreprod.113.116111 24648398

[B94] Yathish H. M. Kumar S. Chaudhary R. Mishra C. Sivakumar A. Kumar A. (2018). Nucleotide variability of protamine genes influencing bull sperm motility variables. *Anim, Reprod. Sci.* 193 126–139. 10.1016/j.anireprosci.2018.04.060 29657074

[B95] Yathish H. M. Kumar S. Dubey P. P. Modi R. P. Chaudhary R. Kumar S. (2017). Profiling of sperm gene transcripts in crossbred (*Bos taurus* x Bos indicus) bulls. *Anim. Reprod. Sci.* 177 25–34. 10.1016/j.anireprosci.2016.12.003 27993430

[B96] Zhao Y. Li Q. Yao C. Wang Z. Zhou Y. Wang Y. (2006). Characterization and quantification of mRNA transcripts in ejaculated spermatozoa of fertile men by serial analysis of gene expression. *Hum. Reprod.* 21 1583–1590. 10.1093/humrep/del027 16501037

[B97] Zhou J. H. Zhou Q. Z. Lyu X. M. Zhu T. Chen Z. J. Chen M. K. (2015). The expression of cysteine-rich secretory protein 2 (CRISP2) and its specific regulator miR-27b in the spermatozoa of patients with asthenozoospermia. *Biol. Reprod.* 92:28. 25505194 10.1095/biolreprod.114.124487

