# Supplementary material for: Comparative Transcriptomic Analysis of Spermatozoa From High- and Low-Fertile Crossbred Bulls: Implications for Fertility Prediction
Source: Front Cell Dev Biol. 2021 May 10;9:647717. doi: 10.3389/fcell.2021.647717 (PMC8141864; doi:10.3389/fcell.2021.647717)
Supplement: Supplementary file 1 [file Data_Sheet_1.docx]

**Supplementary Figures**

**Title of the manuscript:** Comparative transcriptomic analysis of spermatozoa from high- and low-fertile crossbred bulls: Implications for fertility prediction

**Authors:** Mani Arul Prakash, Arumugan Kumaresan, Ebenezer Samuel King John Peter, Pradeep Nag, Ankur Sharma, Manish Kumar Sinha, Elango Kamaraj, Tirtha Kumar Datta

**SUPPLEMENTARY FIGURE 1:** Conception rates of bulls used in the study


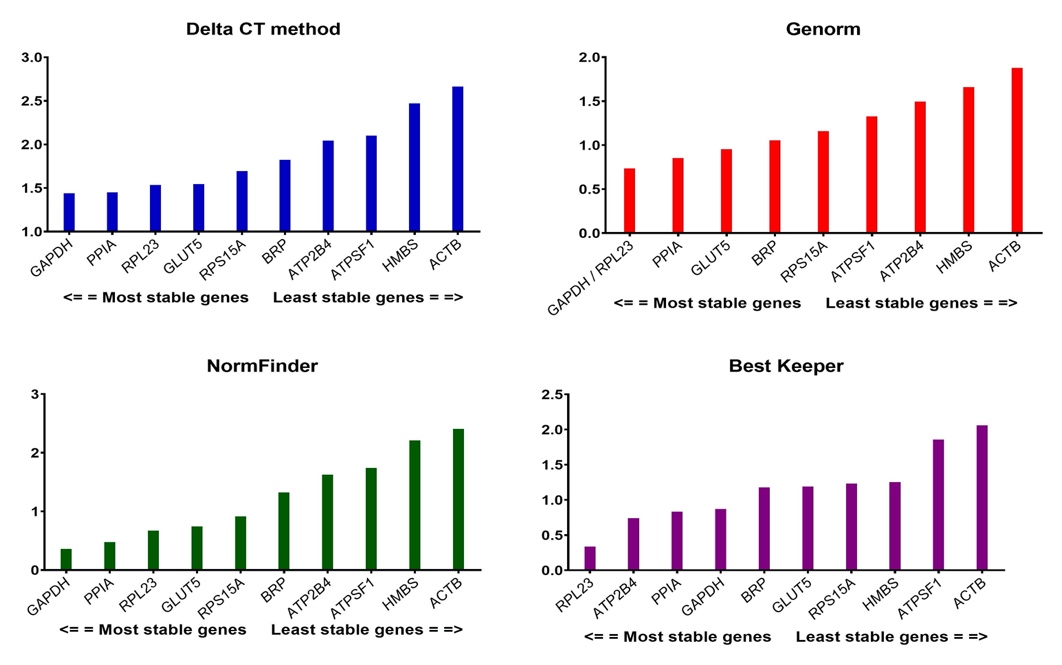


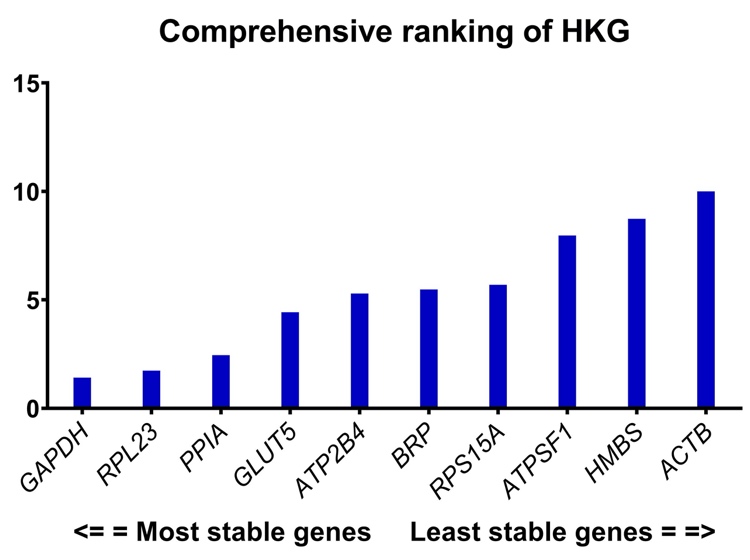


**SUPPLEMENTARY FIGURE 2:** Expression stability of 10 commonly used housekeeping genes as analyzed using Genorm, Normfinder, Delta Ct and the comprehensive ranking by RefFinder

**
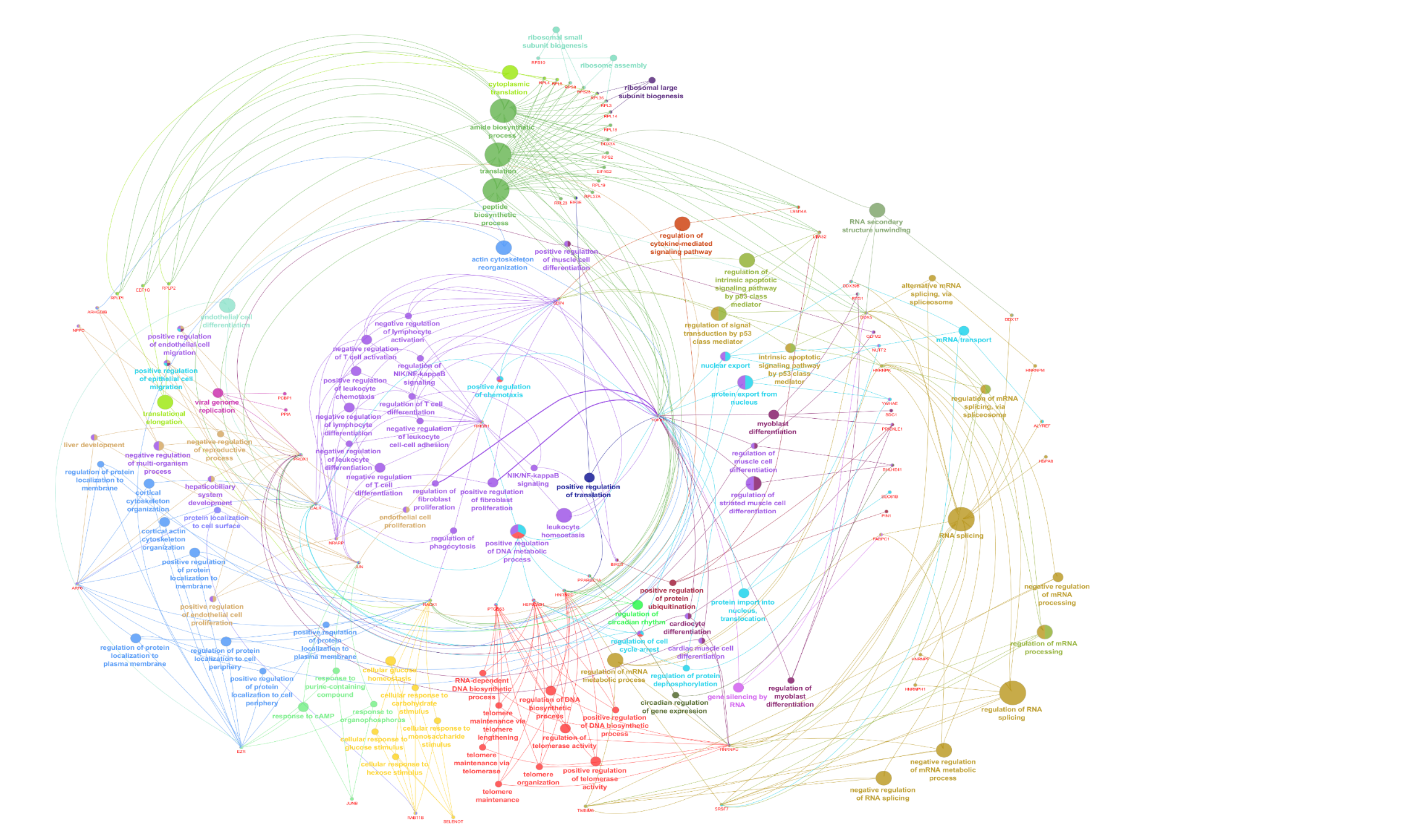
**

**SUPPLEMENTARY FIGURE 3:** Interaction of genes involved in biological process of sperm transcripts upregulated in low fertile bull


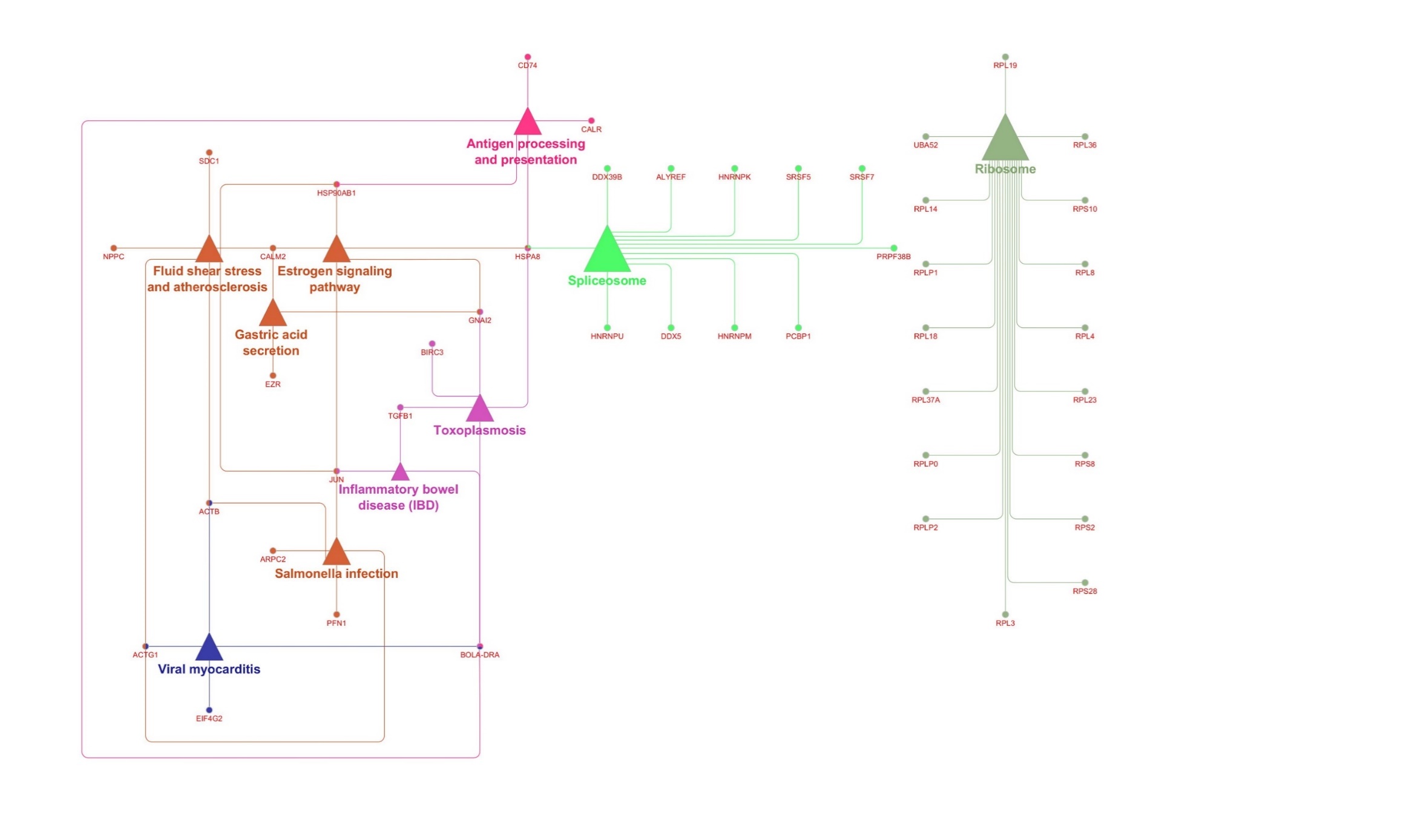


**SUPPLEMENTARY FIGURE 4:** Interaction of genes involved in KEGG pathway of sperm transcripts upregulated in low fertile bull


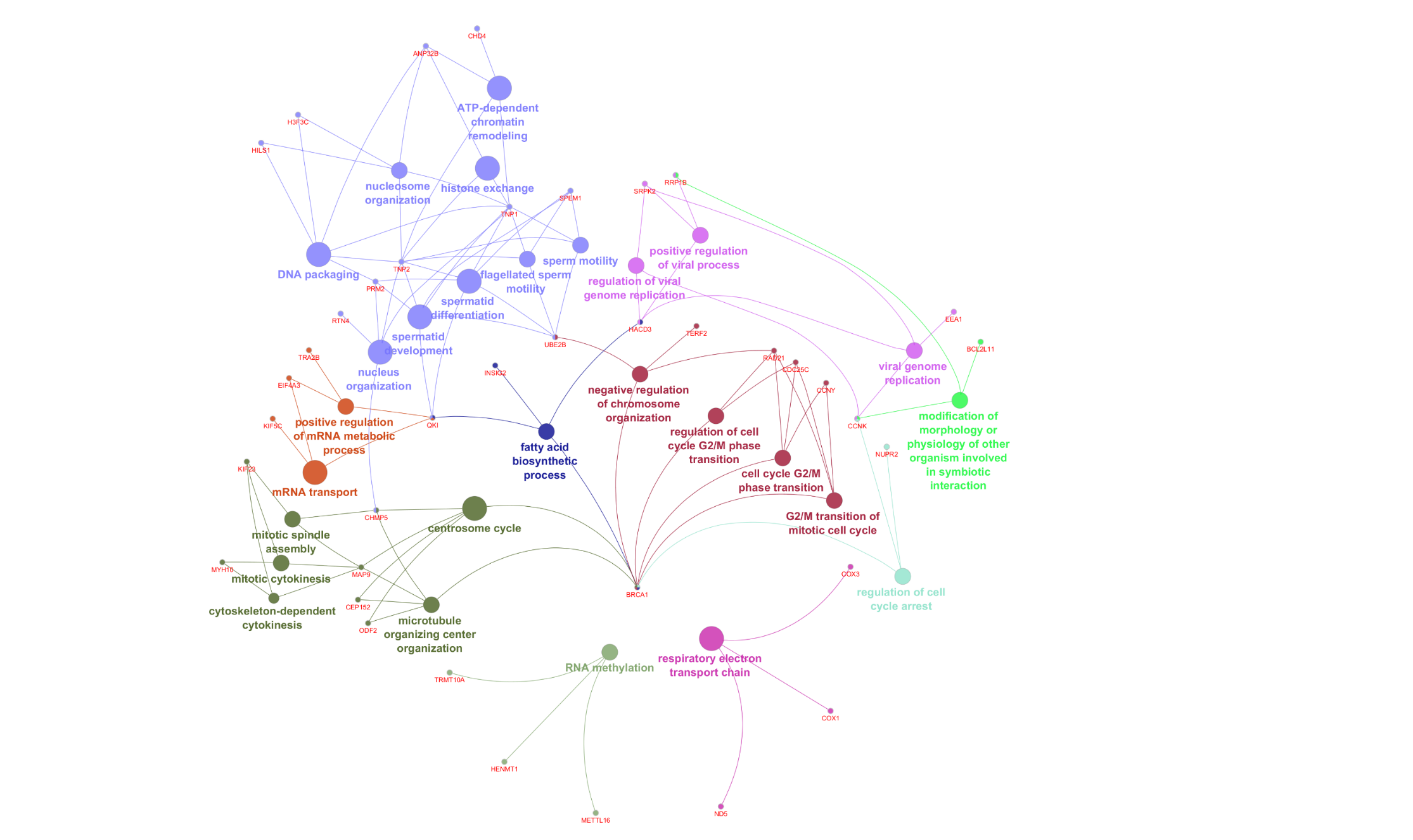


**SUPPLEMENTARY FIGURE 5:** Interaction of genes involved in biological process of sperm transcripts down-regulated in low fertile bull


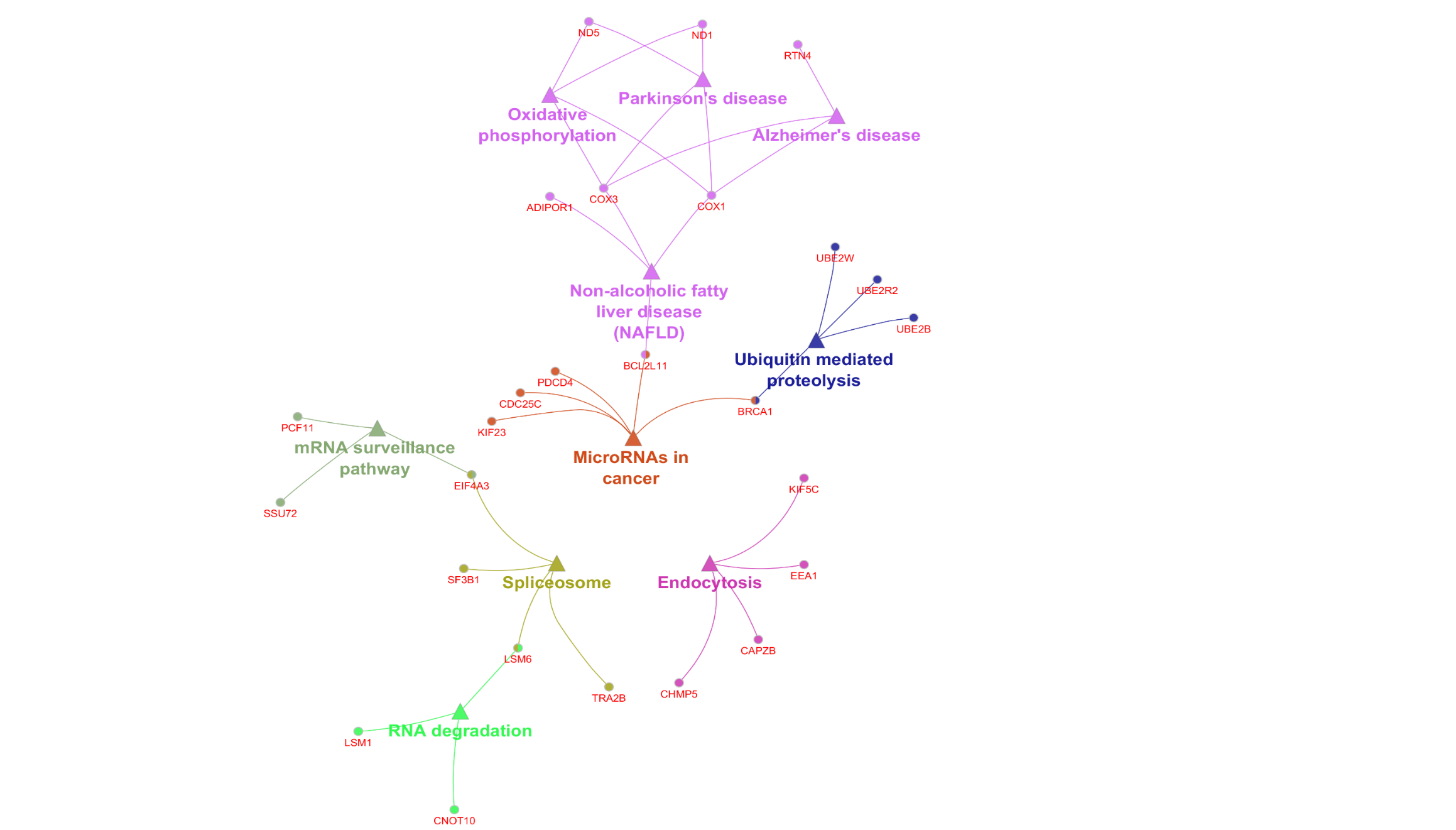


**SUPPLEMENTARY FIGURE 6:** Interaction of genes involved in KEGG pathway of sperm transcripts down-regulated in low fertile bull


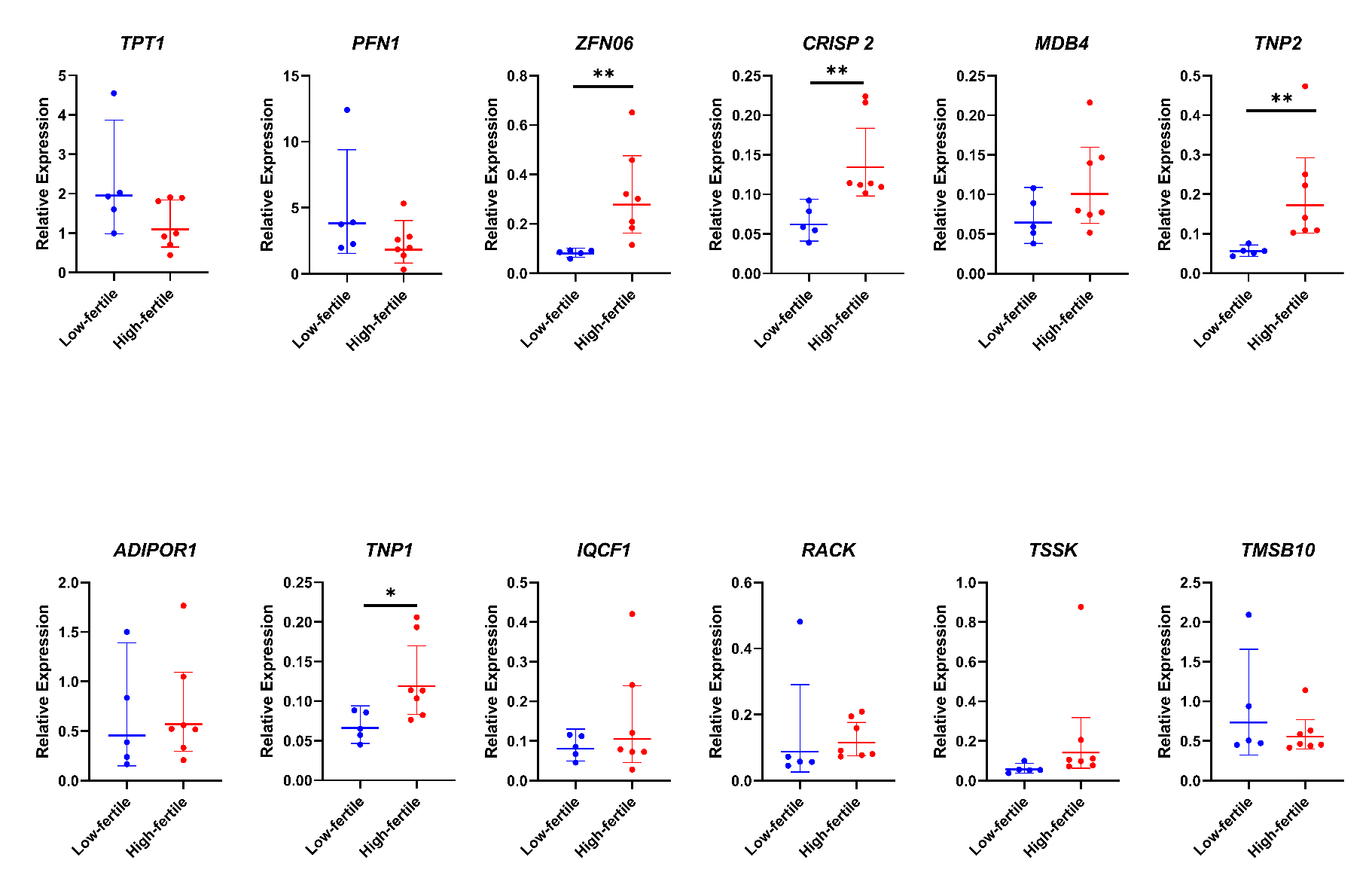


**SUPPLEMENTARY FIGURE 7:** Relative expression of genes in high- and low-fertile crossbred bulls. The geometric mean with 95% Confidence Interval was calculated for high- and low-fertile bulls. Each dot represents an individual bull in a given category.
