# Supplementary material for: Comparative Transcriptomic Analysis of Spermatozoa From High- and Low-Fertile Crossbred Bulls: Implications for Fertility Prediction
Source: Front Cell Dev Biol. 2021 May 10;9:647717. doi: 10.3389/fcell.2021.647717 (PMC8141864; doi:10.3389/fcell.2021.647717)
Supplement: Supplementary file 2 [file Table_1.docx]

**Supplementary Tables**

**Title of the manuscript:** Comparative transcriptomic analysis of spermatozoa from high- and low-fertile crossbred bulls: Implications for fertility prediction

**Authors:** Mani Arul Prakash, Arumugan Kumaresan, Ebenezer Samuel King John Peter, Pradeep Nag, Ankur Sharma, Manish Kumar Sinha, Elango Kamaraj, Tirtha Kumar Datta

**SUPPLEMENTARY TABLE 1: Primers used for Real time quantification**

| **S. No** | **Genes** | **Primer sequence** | **Product size** | **Annealing temperature** | **Accession number** |
| --- | --- | --- | --- | --- | --- |
| 1. | *TPT1* | FP-ACCGGGACCTCATTAGCCA | 106 bp | 60°C | NM_001014388.1 |
|  |  | RP-GTCCTACTGACCATCTTCCCC |  |  |  |
| 2. | *PFN1* | FP-CCTTCGTCAACATCACGCCT | 86 bp | 60°C | NM_001015592.1 |
|  |  | RP-CCCAAGTGTTAGCCCGTTCA |  |  |  |
| 3. | *ZNF706* | FP-TGAGAGAACAAAGGCTGCGAG | 181 bp | 60°C | NM_001199073.1 |
|  |  | RP-GGGTCTGGCATTTGTGTCCTA |  |  |  |
| 4. | *MDB4* | FP-TGGAAGCAGGTACACCCTGAA | 184 bp | 60°C | NM_001034405.2 |
|  |  | RP-CTATACGCCTGGAAAGGTGGT |  |  |  |
| 5. | *TNP2* | FP-ACAGACACACCATGCACTCC | 172 bp | 60°C | NM_174200.1 |
|  |  | RP-TCAGTTGTACTTCCGTCCTGAG |  |  |  |
| 6. | *TNP1* | FP-TGTCGACCAGCCGCAAATTA | 149 bp | 60°C | NM_174199.2 |
|  |  | RP-ATTGCGATTGGCATCGTCAC |  |  |  |
| 7. | *CRISP2* | FP-GCCGCTCTGCAACAGAAGA | 68 bp | 60°C | NM_001038089.1 |
|  |  | RP-AGGAAGAGCATGGTGTTTGGT |  |  |  |
| 8. | *ADIPOR1* | FP-GATCGGCCTGCTGATCATG | 99 bp | 60°C | NC_037343.1 |
|  |  | RP-GACGCAGACGATGGAGAGGTA |  |  |  |
| 9. | RACK1 | FP- ATCTGGGACTTGGAGGGCAA | 172 bp | 60°C | NM_175802.3 |
|  |  | RP- CGATGGTTACCTGCCACACT |  |  |  |
| 10. | IQCF1 | FP- AGGCAGAGGCTCAACAAGAG | 116 bp | 60°C | NM_001075773.1 |
|  |  | RP- TGCTTCTGGATCACTGACGG |  |  |  |
| 11. | TSSK6 | FP- CGACCTCAAGTGCGAAAACG | 107 bp | 60°C | XM_027548402.1 |
|  |  | RP- TGGTGCTCAGATCGGGGTAT |  |  |  |
| 12. | TMSB10 | FP-CTGCCGACCAAAGAGACCA | 129 bp | 60°C | NM_174623.2 |
|  |  | RP- ATCTTGCAGGTGGCTCTTCC |  |  |  |
| 13. | *GAPDH* | FP-CTGAGGACCAGGTTGTCTCCTG | 141 bp | 60°C | NM_001034034.1 |
|  |  | RP-CCCTGTTGCTGTAGCCAAATTC |  |  |  |

**SUPPLEMENTARY TABLE 2:** List of housekeeping genes used in the study and their annealing temperature

| Gene Name | Primer Pair 5’-3’ | Amplicon size (bp) | Annealing Temperature | Accession No. |
| --- | --- | --- | --- | --- |
| *ATPSF1* | F-TTGCTGTGTGAACGCTACCT | 85 | 60 | NM_001038501.2 |
|  | R-CTGCGTTCTTCAGAGAGGGG |  |  |  |
| *GLUT5* | F-AGCTAAAGGAGTTTCCACCGT | 207 | 60 | NM_001101042.2 |
|  | R-GGTGGTAGGTCAATCCGGG |  |  |  |
| *RPL23* | F-CAGCGGTGGTAATTCGACAAC | 116 | 60 | NM_001035014.2 |
|  | R-GGCGGAACCTTTCATCTCG |  |  |  |
| *RPS15A* | F-AATGGTGCGCATGAATGTC | 100 | 60 | XM_005679050 |
|  | R-GACTTTGGAGCACGGCCTAA |  |  |  |
| *HMBS* | F-GTTCGAGCCAAAGACCAGGA | 170 | 60 | NM_001046207.1 |
|  | R-CCAGTCAGGTACAGTTGCCC |  |  |  |
| *ATP2B4* | F-GTTTGGCTGCACAGTGATGG | 143 | 60 | NM_001172594 |
|  | R-TCCCAGATAGGCCTTCCACA |  |  |  |
| *PPIA* | F-ATGCTGGCCCCAACACAA | 101 | 60 | XM_001252921.1 |
|  | R-CCCTCTTTCACCTTGCCAAA |  |  |  |
| *BRP* | F-CCAGGCTTTAGGCATCACCA | 94 | 60 | NM_001012682.1 |
|  | R-GGCGCCTACTTTGTCTCCTGT |  |  |  |
| *GAPDH* | F-CTGAGGACCAGGTTGTCTCCTG | 141 | 60 | NM_001034034.1 |
|  | R-CCCTGTTGCTGTAGCCAAATTC |  |  |  |
| *ACTB* | F-CTCTTCCAGCCTTCCTTCCT | 118 | 60 | NM_173979 |
|  | R- ACAGCACTGTGTTGGCGTAG |  |  |  |

**SUPPLEMENTARY TABLE 3:** Gene Ontology (GO) analysis of sperm transcripts upregulated in low fertile bull

| **Gene Ontology Terms** | **Count** | **Transcripts/genes** |
| --- | --- | --- |
| **Biological process (BP)** | | |
| GO:0006412~translation | 12 | RPL14, RPL18, RPL23, RPL3, RPL36, RPL37A, RPL4, RPS2, RPS28, RPS8, SLC25A6, UBA52 |
| GO:0045944~positive regulation of transcription from RNA polymerase II promoter | 11 | DDX3X, DDX5, JUNB, PPARGC1A, JUN, HMGB1, PIN1, PCBP1, PROX1, TGFB1, TNFSF8 |
| GO:0098609~cell-cell adhesion | 9 | RAB11B, EIF3E, HSPA8, HNRNPK, PCBP1, RACK1, RPS2, TAGLN2, YWHAE, |
| GO:0000122~negative regulation of transcription from RNA polymerase II promoter | 9 | DDX5, JUN, JUNB, NRARP, CALR, EZR, PROX1, TGFB1, ZBTB18, |
| GO:0008380~RNA splicing | 6 | ALYREF, HSPA8, HNRNPF, HNRNPK, PABPC1, SRSF7 |
| GO:0006397~mRNA processing | 6 | ALYREF, HSPA8, HNRNPF, HNRNPK, PABPC1, SRSF7, |
| GO:0002181~cytoplasmic translation | 5 | RPL36, RPL8, RPLP0, RPLP1, RPLP2, |
| GO:0010501~RNA secondary structure unwinding | 4 | DDX17, DDX3X, DDX5, DDX39B, |
| GO:0051726~regulation of cell cycle | 4 | JUN, JUNB, HSPA8, RACK1, |
| GO:0006457~protein folding | 4 | ACTB, CALR, HSP90AB1, PPIA, |
| GO:0070374~positive regulation of ERK1 and ERK2 cascade | 4 | CD74, JUN, HMGB1, TGFB1, |
| GO:0006414~translational elongation | 3 | EEF1G, RPLP1, RPLP2, |
| GO:0006611~protein export from nucleus | 3 | CALR, NUTF2, TGFB1, |
| GO:0045070~positive regulation of viral genome replication | 3 | DDX3X, PPIA, PABPC1, |
| GO:0000060~protein import into nucleus, translocation | 3 | SEC61B, NUTF2, TGFB1, |
| GO:0050714~positive regulation of protein secretion | 3 | EZR, PPIA, TGFB1, |
| GO:0051028~mRNA transport | 3 | ALYREF, NUTF2, SRSF7, |
| GO:0006413~translational initiation | 3 | DDX3X, EIF3E, EIF4G2 |
| GO:0021762~substantia nigra development | 3 | ACTB, CALM2, YWHAE |
| GO:0042752~regulation of circadian rhythm | 3 | PPARGC1A, HNRNPD, PROX1 |
| GO:0048146~positive regulation of fibroblast proliferation | 3 | CD74, JUN, TGFB1 |
| GO:0009615~response to virus | 3 | DDX3X, EEF1G, STMN1 |
| GO:0032922~circadian regulation of gene expression | 3 | PPARGC1A, BHLHE41, HNRNPU |
| GO:0001938~positive regulation of endothelial cell proliferation | 3 | JUN, NRARP, PROX1 |
| GO:0001934~positive regulation of protein phosphorylation | 3 | PIN1, RACK1, TGFB1 |
| GO:0010468~regulation of gene expression | 3 | DDX3X, DDX39B, TENT5A |
| GO:0039694~viral RNA genome replication | 2 | PCBP2, PCBP1 |
| GO:0045581~negative regulation of T cell differentiation | 2 | CD74, NRARP |
| GO:0051343~positive regulation of cyclic-nucleotide phosphodiesterase activity | 2 | CALM2, RACK1 |
| GO:1990441~negative regulation of transcription from RNA polymerase II promoter in response to endoplasmic reticulum stress | 2 | JUN, TMBIM6 |
| GO:0010941~regulation of cell death | 2 | JUN, JUNB |
| GO:0051085~chaperone mediated protein folding requiring cofactor | 2 | CD74, HSPA8 |
| GO:0007184~SMAD protein import into nucleus | 2 | JUN, TGFB1 |
| GO:0031953~negative regulation of protein autophosphorylation | 2 | ERRFI1, JUN |
| GO:0017015~regulation of transforming growth factor beta receptor signaling pathway | 2 | NREP, TGFB1 |
| GO:1901224~positive regulation of NIK/NF-kappaB signaling | 2 | CALR, HMGB1 |
| GO:0051591~response to cAMP | 2 | JUN, JUNB |
| GO:0009612~response to mechanical stimulus | 2 | JUN, JUNB |
| GO:2000249~regulation of actin cytoskeleton reorganization | 2 | ARHGDIB, TGFB1 |
| **Molecular Functions (MF)** | | |
| GO:0044822~poly(A) RNA binding | 35 | DDX5, DDX39B, JUN, LSM14A, SEC61B, EIF3E, EZR, TENT5A, FUBP1, HSPA8, HNRNPAB, HNRNPD, HNRNPF, HNRNPH1, HNRNPK, HNRNPU, HMGN2, PPIA, PABPC1, PCBP2, PRPF38B, PKM, RACK1, RPL14, RPL23, RPL36, RPL37A, RPL8, RPS28, RPS8, RPLP0, SRSF5, SRSF7, SRRM2, YWHAE, |
| GO:0003735~structural constituent of ribosome | 17 | RPL14, RPL18, RPL23, RPL3, RPL36, RPL37A, RPL4, RPL8, RPS10, RPS2, RPS28, RPS8, RPLP0, RPLP1, RPLP2, SLC25A6, UBA52 |
| GO:0000166~nucleotide binding | 11 | ALYREF, PPARGC1A, RBM43, HNRNPAB, HNRNPD, HNRNPF, HNRNPH1, HNRNPM, PABPC1, SRSF5, SRSF7 |
| GO:0098641~cadherin binding involved in cell-cell adhesion | 10 | RAB11B, EIF3E, EZR, HSPA8, HNRNPK, PCBP1, RACK1, RPS2, TAGLN2, YWHAE |
| GO:0003723~RNA binding | 9 | ALYREF, PPARGC1A, EIF4G2, FUBP1, HNRNPM, PCBP1, PSMA6, RPL18, RPS28 |
| GO:0070180~large ribosomal subunit rRNA binding | 4 | RPL23, RPLP0, RPLP1, RPLP2 |
| GO:0004004~ATP-dependent RNA helicase activity | 4 | DDX17, DDX3X, DDX5, DDX39B |
| GO:0031625~ubiquitin protein ligase binding | 4 | ACTG1, HSPA8, TMBIM6, YWHAE |
| GO:0003713~transcription coactivator activity | 4 | DDX5, JUN, JUNB, PPARGC1A |
| GO:0003727~single-stranded RNA binding | 3 | LSM14A, HNRNPF, HMGB1 |
| GO:0003705~transcription factor activity, RNA polymerase II distal enhancer sequence-specific binding | 3 | JUN, BHLHE41, PROX1 |
| GO:0001948~glycoprotein binding | 3 | CALR, SDC1, TGFB1 |
| GO:0051082~unfolded protein binding | 3 | CALR, HSP90AB1, HSPA8 |
| GO:0050815~phosphoserine binding | 2 | PIN1, YWHAE |
| GO:0023026~MHC class II protein complex binding | 2 | HSPA8, YWHAE |
| GO:0008143~poly(A) binding | 2 | DDX3X, PABPC1 |
| **Cellular Components (CC)** | | |
| GO:0070062~extracellular exosome | 45 | ARF6, CD74, CTDSPL, DDX3X, DDX5, GNAI2, RAB11B, ARHGDIB, ACTB, ACTG1, ARPC2, CALM2, CALR, COX4I1, EEF1G, EIF3E, EZR, HSPA8, HNRNPD, HNRNPK, BOLA-DRA, MGAT1, NUTF2, PPIA, PABPC1, PCBP1, PCBP2, PSMA6, PKM, RACK1, RPL14, RPL23, RPL37A, RPS2, RPS28, RPS8, RPLP0, RPLP1, SRSF7, STMN1, SDC1, TAGLN2, YWHAE, TPST2, UBA52, |
| GO:0005634~nucleus | 37 | BTG1, DDX3X, ERG, H3F3B, POU2AF1, PPARGC1A, SET, SIX6, NSD3, ACTG1, BIRC3, BHLHE41, CALM2, CALR, COX4I1, EEF1G, EIF3E, HSPA8, HNRNPU, HMGN2, IRF2BP2, OLFM2, OAZ1, PPIA, PROX1, PSMA6, PTMA, PKM, RACK1, RPL37A, RPS8, RPLP0, SLC25A6, TGFB1, UBA52, UBC, ZBTB18, |
| GO:0016020~membrane | 23 | DDX5, EEF1G, EIF3E, HNRNPF, HNRNPH1, HNRNPK, HNRNPU, LTB, PNKD, PPIA, PABPC1, PCBP1, PCBP2, RPL14, RPL18, RPL23, RPL36, RPL8, RPS2, RPS8, RPLP0, SDC1, YWHAE, |
| GO:0005925~focal adhesion | 21 | ARF6, ACTB, ACTG1, ARPC2, CALR, EZR, HSPA8, HNRNPK, PPIA, PABPC1, PCBP2, RPL18, RPL23, RPL37A, RPL8, RPS2, RPS8, RPLP0, RPLP1, SDC1, YWHAE, |
| GO:0005654~nucleoplasm | 17 | DDX5, GNAI2, JUN, JUNB, BIRC3, FUBP1, HNRNPAB, HNRNPD, HNRNPF, HNRNPH1, HNRNPK, HNRNPU, NUTF2, PCBP1, PCBP2, RPS2, SRSF7, |
| GO:0022625~cytosolic large ribosomal subunit | 11 | RPL14, RPL18, RPL23, RPL3, RPL36, RPL37A, RPL4, RPL8, RPLP0, RPLP1, RPLP2, |
| GO:0005913~cell-cell adherens junction | 10 | RAB11B, EIF3E, EZR, HSPA8, HNRNPK, PCBP1, RACK1, RPS2, TAGLN2, YWHAE |
| GO:0005730~nucleolus | 10 | DDX5, EZR, HSPA8, RPL18, RPL23, RPL3, RPL36, RPL8, RPS10, SRSF5 |
| GO:0030529~intracellular ribonucleoprotein complex | 9 | ERG, HSPA8, HNRNPAB, HNRNPD, HNRNPU, PABPC1, PCBP1, RPS8, RPLP0 |
| GO:0071013~catalytic step 2 spliceosome | 7 | DDX5, HNRNPF, HNRNPH1, HNRNPK, HNRNPU, PABPC1, SRRM2 |
| GO:0043209~myelin sheath | 7 | ARF6, ACTB, ACTG1, EZR, HSPA8, PKM, UBA52 |
| GO:0022627~cytosolic small ribosomal subunit | 5 | DDX3X, RPS10, RPS2, RPS28, RPS8 |
| GO:0031012~extracellular matrix | 5 | ACTG1, HSPA8, HNRNPK, PKM, SLC25A6 |
| GO:0016607~nuclear speck | 5 | ALYREF, DDX39B, PIN1, SRSF5, SRRM2 |
| GO:0000790~nuclear chromatin | 5 | JUN, JUNB, ACTB, HNRNPK, HMGB1 |
| GO:0036464~cytoplasmic ribonucleoprotein granule | 4 | DDX3X, ACTB, HNRNPU, RPLP0 |
| GO:0005681~spliceosomal complex | 4 | ALYREF, DDX39B, HSPA8, HNRNPF |
| GO:0072562~blood microparticle | 4 | ACTB, ACTG1, HSPA8, TGFB1 |
| GO:0019013~viral nucleocapsid | 3 | HNRNPAB, HNRNPD, HNRNPU |
| GO:0030687~preribosome, large subunit precursor | 3 | RPLP0, RPLP1, RPLP2 |
| GO:0010494~cytoplasmic stress granule | 3 | DDX3X, LSM14A, PABPC1 |
| GO:0005840~ribosome | 3 | RPL18, RPLP0, UBA52 |
| GO:0097433~dense body | 2 | ACTB, ACTG1 |
| GO:0005852~eukaryotic translation initiation factor 3 complex | 2 | DDX3X, EIF3E |
| **Kegg pathway** | | |
| bta03010:Ribosome | 17 | ENSBTAG00000018987, RPL14, RPL18, RPL23, RPL3, RPL36, RPL37A, RPL4, RPL8, RPS10, RPS2, RPS28, RPS8, RPLP0, RPLP1, RPLP2, UBA52 |
| bta03040:Spliceosome | 11 | ALYREF, DDX5, DDX39B, HSPA8, HNRNPK, HNRNPM, HNRNPU, PCBP1, PRPF38B, SRSF5, SRSF7 |
| bta05168:Herpes simplex infection | 7 | ALYREF, CD74, JUN, HNRNPK, BOLA-DRA, SRSF5, SRSF7 |
| bta05164:Influenza A | 6 | DDX39B, JUN, ACTB, ACTG1, HSPA8, BOLA-DRA |
| bta05205:Proteoglycans in cancer | 6 | DDX5, ACTB, ACTG1, EZR, SDC1, TGFB1 |
| bta04612:Antigen processing and presentation | 5 | CD74, CALR, HSP90AB1, HSPA8, BOLA-DRA |
| bta05132:Salmonella infection | 5 | JUN, ACTB, ACTG1, ARPC2, PFN1 |
| bta04915:Estrogen signaling pathway | 5 | GNAI2, JUN, CALM2, HSP90AB1, HSPA8 |
| bta05145:Toxoplasmosis | 5 | GNAI2, BIRC3, HSPA8, BOLA-DRA, TGFB1 |
| bta04921:Oxytocin signaling pathway | 5 | GNAI2, JUN, ACTB, ACTG1, CALM2 |
| bta05416:Viral myocarditis | 4 | ACTB, ACTG1, EIF4G2, BOLA-DRA |
| bta05323:Rheumatoid arthritis | 4 | JUN, LTB, BOLA-DRA, TGFB1 |

**SUPPLEMENTARY TABLE 4:** Gene Ontology (GO) analysis of sperm transcripts down regulated in low fertile bull

| **Gene Ontology Terms** | **Count** | **Transcripts/genes** |
| --- | --- | --- |
| **Biological Process (BP)** | | |
| GO:0007275~multicellular organism development | 9 | QKI, ODF1, TNP1, PRM2, CFDP1, TNP2, ODF2, SPEM1, MEA1 |
| GO:0007283~spermatogenesis | 7 | ODF1, BCL2L11, PRM2, TNP2, ODF2, SPEM1, MEA1 |
| GO:0030154~cell differentiation | 6 | QKI, ODF1, TNP2, ODF2, SPEM1, MEA1 |
| GO:0001701~in utero embryonic development | 5 | YBX1, UBE2B, BCL2L11, MYH10, RBBP6 |
| GO:1902600~hydrogen ion transmembrane transport | 4 | MT-CYB, MT-CO2, COX3, COX1 |
| GO:0008380~RNA splicing | 4 | YBX1, QKI, EIF4A3, LSM1 |
| GO:0006397~mRNA processing | 4 | YBX1, QKI, EIF4A3, LSM1 |
| GO:0008360~regulation of cell shape | 4 | CSNK1G2, CCL11, CFDP1, MYH10 |
| GO:0006281~DNA repair | 4 | UBE2B, UBE2W, BRCA1, MBD4 |
| GO:0042773~ATP synthesis coupled electron transport | 3 | MT-CO2, ND5, MT-ND4 |
| GO:0006513~protein monoubiquitination | 3 | UBE2B, UBE2W, UBE2R2 |
| GO:0051028~mRNA transport | 3 | QKI, KIF5C, EIF4A3 |
| GO:0090036~regulation of protein kinase C signaling | 2 | CAPZB, AKAP12 |
| GO:0006369~termination of RNA polymerase II transcription | 2 | SSU72, PCF11 |
| GO:0090084~negative regulation of inclusion body assembly | 2 | DNAJB8, DNAJA4 |
| GO:0045717~negative regulation of fatty acid biosynthetic process | 2 | BRCA1, INSIG2 |
| GO:0006301~postreplication repair | 2 | UBE2B, BRCA1 |
| **Molecular Functions (MF)** | | |
| GO:0003677~DNA binding | 15 | BRCA1, DNAJC2, SETDB2, YBX2, BAZ2B, CHD4, HMGB4, HILS1, MBD4, NUCB1, PRM2, SSRP1, ELOA, TNP1, TNP2 |
| GO:0044822~poly(A) RNA binding | 15 | AKAP1, DNAJC2, LSM1, LSM6, PDAP1, RBBP6, RTF1, SRPK2, YBX1, EIF1, MRPL40, RTN4, RRP1B, SSRP1, TRMT10A, |
| GO:0003729~mRNA binding | 5 | PCF11, QKI, EIF4A3, SF3B1, TRA2B |
| GO:0008137~NADH dehydrogenase (ubiquinone) activity | 4 | ND1, MT-ND2, MT-ND4, ND5 |
| GO:0004129~cytochrome-c oxidase activity | 3 | COX1, MT-CO2, COX3 |
| GO:0051015~actin filament binding | 3 | CAPZB, MYH10, PICK1 |
| GO:0048487~beta-tubulin binding | 2 | CAPZB, IFT74 |
| GO:0016538~cyclin-dependent protein serine/threonine kinase regulator activity | 2 | CCNK, CCNY |
| **Cellular Components (CC)** | | |
| GO:0005634~nucleus | 50 | BRCA1, DNAJB8, DNAJC2, FUNDC2, HYLS1, LSM1, LYRM4, QKI, SETDB2, THAP1, ANP32B, BAZ2B, CABYR, CSNK1G2, CDC25C, CHMP5, CHD4, CCNK, CCNY, ENSBTAG00000010813, DYNLL2, EIF1, FAM71B, FAM71D, FAM71F1, HSPB9, HMGB4, H3F3C, HILS1, IFT74, LUZP1, MBD4, METTL16, MYH10, MTPN, NHLH2, NUPR2, ODF1, ODF2, PRM2, PPM1A, RRP1B, SSRP1, TRMT10A, ELOA, TNP1, UBE2W, MGC140080, ZNF24, ZNF706 |
| GO:0005737~cytoplasm | 40 | AKAP12, BRCA1, DCAF12, DDHD1, HYLS1, PCF11, QKI, SRPK2, SSU72, ANP32B, ATG4D, CABYR, CSNK1G2, CDC25C, CHD4, ENSBTAG00000010813, DYNLL2, DBIL5, EIF1, EIF4A3, FAM71D, GSTO2, GAP43, HSPB9, HIPK1, KIF5C, LELP1, LYPLA1, MTPN, PICK1, SPEM1, SSRP1, SOCS7, THTPA, TUFT1, UBE2B, UBE2R2, UBE2W, ZMYND19, ZNF706, |
| GO:0016020~membrane | 15 | CNOT10, DNAJA4, ORAI3, RAD21, CAPZB, CSNK1G2, CHD4, MT-CO2, DYNLL2, EIF4A3, IRGC, LUZP1, NUCB1, PPM1A, ZMYND19, |
| GO:0005739~mitochondrion | 14 | HACD3, AKAP1, MT-ATP6, BCL2L11, DCAF5, LYRM4, MT-ND4, ND5, ACSBG2, APOPT1, MT-CYB, MT-CO2, COX3, MYH10 |
| GO:0005743~mitochondrial inner membrane | 8 | MT-ATP6, ND1, MT-ND2, ND5, CLPX, MT-CYB, COX1, MT-CO2 |
| GO:0005813~centrosome | 7 | DCAF12, HYLS1, CEP152, CHD4, DYNLL2, IFT74, KIF23 |
| GO:0070469~respiratory chain | 5 | MT-ND2, ND5, MT-CYB, COX1, MT-CO2 |
| GO:0000786~nucleosome | 5 | H3F3C, HILS1, PRM2, TNP1, TNP2 |
| GO:0005747~mitochondrial respiratory chain complex I | 4 | ND1, MT-ND2, MT-ND4, ND5 |
| GO:0045277~respiratory chain complex IV | 3 | COX1, MT-CO2, COX3 |
| GO:0005694~chromosome | 3 | BRCA1, SETDB2, HMGB4 |
| GO:0008290~F-actin capping protein complex | 2 | CAPZB, MTPN |
| **Kegg pathway** | | |
| bta00190:Oxidative phosphorylation | 9 | MT-ATP6, ND1, MT-ND2, MT-ND4, ND5, MT-CYB, COX1, MT-CO2, COX3, |
| bta05012:Parkinson's disease | 9 | MT-ATP6, ND1, MT-ND2, MT-ND4, ND5, MT-CYB, COX1, MT-CO2, COX3 |
| bta04932:Non-alcoholic fatty liver disease (NAFLD) | 6 | BCL2L11, ADIPOR1, MT-CYB, COX1, MT-CO2, COX3, |
| bta05010:Alzheimer's disease | 5 | MT-ATP6, MT-CYB, COX1, MT-CO2, COX3 |
| bta05016:Huntington's disease | 5 | MT-ATP6, MT-CYB, COX1, MT-CO2, COX3 |
| bta04260:Cardiac muscle contraction | 4 | MT-CYB, COX1, MT-CO2, COX3, |
| bta03040:Spliceosome | 4 | LSM6, EIF4A3, SF3B1, TRA2B, |
| bta04120:Ubiquitin mediated proteolysis | 4 | BRCA1, UBE2B, UBE2R2, UBE2W, |

**SUPPLEMENTARY TABLE 5:** Gene Ontology (GO) analysis of sperm transcripts unique to high fertile bull

| **Gene Ontology Terms** | **Count** | **Transcripts/genes** |
| --- | --- | --- |
| **Biological Process (BP)** | | |
| GO:0045087~innate immune response | 3 | PYCARD, APCS, ENSBTAG00000039963 |
| **Cellular Components (CC)** | | |
| - | - | - |
| **Molecular Functions (MF)** | | |
| GO:0046961~proton-transporting ATPase activity, rotational mechanism | 2 | ATP5F1D, ATP6V1C1 |
| GO:0061631~ubiquitin conjugating enzyme activity | 2 | UBE2J1, CDC34 |
| **Kegg pathway** | | |
| bta05016:Huntington's disease | 4 | ATP5F1D, CLTA, MGC148714, TFAM |
| bta00190:Oxidative phosphorylation | 3 | ATP5F1D, ATP6V1C1, MGC148714 |
| bta05012:Parkinson's disease | 3 | ATP5F1D, MGC148714, UBE2J1 |

**SUPPLEMENTARY TABLE 6:** Gene Ontology (GO) analysis of sperm transcripts unique to high fertile bull

| **Gene Ontology Terms** | **Count** | **Transcripts/genes** |
| --- | --- | --- |
| **Biological Process (BP)** | | |
| GO:0006412~translation | 39 | RPL21, RPL38, RPS20, RPL11, RPSA, RPS3, RPL10A, RPL34, RPL13A, RPS23, RPL27A, RPL32, EEF2, RPS27, RPS18, RPS17, RPL37, RPL10, RPL28, RPS14, RPS5, ENSBTAG00000019007, RPL24, MRPL13, RPS12, RPS15, ENSBTAG00000040435, RPS27A, RPL30, RPS21, ENSBTAG00000033887, RPL7A, RPL18A, RPL27, RPS7, RPS11, RPS9, RPL5, RPS24, , |
| GO:0000028~ribosomal small subunit assembly | 6 | RPS15, ENSBTAG00000040435, RPS27, RPS17, RPSA, RPS5 |
| GO:0002181~cytoplasmic translation | 5 | RPL7, RPL29, RPL35A, RPL26, RPL31 |
| GO:0000027~ribosomal large subunit assembly | 4 | RPL24, RPL11, RPL10, RPL5 |
| GO:0098609~cell-cell adhesion | 4 | RPL24, EEF2, RPL29, RPL7A |
| GO:0000398~mRNA splicing, via spliceosome | 4 | U2AF1, ZMAT2, SYF2, HNRNPA2B1 |
| GO:0006364~rRNA processing | 3 | RPL11, RPL26, RPS7 |
| GO:0008380~RNA splicing | 3 | RBM8A, C1QBP, SRSF3 |
| GO:0000461~endonucleolytic cleavage to generate mature 3'-end of SSU-rRNA from (SSU-rRNA, 5.8S rRNA, LSU-rRNA) | 2 | RPSA, RPS21 |
| GO:0000447~endonucleolytic cleavage in ITS1 to separate SSU-rRNA from 5.8S rRNA and LSU-rRNA from tricistronic rRNA transcript (SSU-rRNA, 5.8S rRNA, LSU-rRNA) | 2 | RPSA, RPS21, |
| GO:0032695~negative regulation of interleukin-12 production | 2 | C1QBP, ACP5 |
| GO:0006414~translational elongation | 2 | EEF1B2, EEF2 |
| **Cellular Components (CC)** | | |
| GO:0070062~extracellular exosome | 42 | ARL8A, ATP5F1B, NDUFA13, ACP5, ARPC3, B2M, CORO1A, DBI, EEF2, HSPB1, HSPE1, HNRNPA2B1, LCP1, MYL6, KCTD12, PSME1, PSMB9, PDIA3, RHOA, RAC1, RPL10A, RPL11, RPL24, RPL26, RPL28, RPL30, RPL34, RPL7, RPL7A, RPS11, RPS18, RPS20, RPS25, RPS27A, RPS3, RPS5, RPS7, RPS9, SARS, SNX2, TUBB, YWHAG |
| GO:0016020~membrane | 29 | ARPC3, DYNLRB1, HSPE1, HNRNPA2B1, MYL6, RAC1, RPL10A, RPL11, RPL18A, RPL24, RPL26, RPL28, RPL29, RPL30, RPL32, RPL7, RPL7A, RPS11, RPS12, RPS18, RPS20, RPS23, RPS24, RPS27A, RPS5, RPS7, RPS9, RPSA, YWHAG |
| GO:0022625~cytosolic large ribosomal subunit | 21 | RPL10, RPL10A, RPL11, RPL13A, RPL18A, RPL24, RPL26, RPL27, RPL27A, RPL28, RPL29, RPL30, RPL31, RPL32, RPL34, RPL35A, RPL37, RPL38, RPL5, RPL7, RPL7A, |
| GO:0022627~cytosolic small ribosomal subunit | 19 | ENSBTAG00000019007, ENSBTAG00000040435, RPS11, RPS12, RPS15, RPS17, RPS18, RPS20, RPS21, RPS23, RPS24, RPS25, RPS27, RPS27A, RPS3, RPS5, RPS7, RPS9, RPSA |
| GO:0005925~focal adhesion | 18 | ARPC3, B2M, HSPB1, LCP1, PDIA3, RHOA, RAC1, RPL10A, RPL30, RPL7, RPL7A, RPS11, RPS18, RPS3, RPS5, RPS7, RPS9, YWHAG |
| GO:0005730~nucleolus | 15 | MKNK2, C1QBP, RPL10A, RPL11, RPL34, RPL5, RPL7, RPL7A, RPS11, RPS23, RPS25, RPS27A, RPS3, RPS7, RPS9 |
| GO:0031012~extracellular matrix | 12 | ATP5F1B, EEF2, MYL6, RPL11, RPL30, RPS11, RPS18, RPS20, RPS3, RPS5, RPS7, TUBB |
| GO:0005829~cytosol | 12 | GABARAPL2, ARPC3, C1QBP, CORO1A, LCP1, MYL6, PFDN2, RHOA, RAC1, RPS18, SNX2, SAT1 |
| GO:0005840~ribosome | 9 | ENSBTAG00000033887, MRPL13, RPL10, RPL21, RPL27A, RPL34, RPS14, RPS17, RPS25 |
| GO:0030027~lamellipodium | 5 | ARPC3, CORO1A, RHOA, RAC1, SNX2 |
| GO:0005759~mitochondrial matrix | 5 | LYRM7, C1QBP, HSPE1, RPS3, SOD2 |
| GO:0030529~intracellular ribonucleoprotein complex | 4 | HNRNPA2B1, HNRNPC, RPS3, RPS9 |
| GO:0005913~cell-cell adherens junction | 4 | EEF2, RPL24, RPL29, RPL7A |
| GO:0016607~nuclear speck | 4 | RBM8A, U2AF1, DUSP11, SRSF3 |
| GO:0043209~myelin sheath | 4 | ATP5F1B, PDIA3, RPS27A, YWHAG |
| GO:0001891~phagocytic cup | 3 | CORO1A, LCP1, RAC1 |
| GO:0036464~cytoplasmic ribonucleoprotein granule | 3 | RAC1, RPL28, TUBB |
| GO:0005884~actin filament | 3 | CORO1A, LCP1, RAC1 |
| GO:0030686~90S preribosome | 2 | RPS7, RPSA |
| GO:0042612~MHC class I protein complex | 2 | JSP.1, B2M |
| **Molecular Functions (MF)** | | |
| GO:0003735~structural constituent of ribosome | 42 | ENSBTAG00000019007, ENSBTAG00000040435, ENSBTAG00000033887, MRPL13, RPL10, RPL10A, RPL11, RPL13A, RPL18A, RPL21, RPL24, RPL26, RPL27, RPL27A, RPL28, RPL29, RPL30, RPL31, RPL32, RPL34, RPL35A, RPL37, RPL38, RPL5, RPL7, RPS11, RPS12, RPS14, RPS15, RPS17, RPS18, RPS20, RPS21, RPS23, RPS24, RPS27, RPS27A, RPS3, RPS5, RPS7, RPS9, RPSA |
| GO:0044822~poly(A) RNA binding | 36 | SYF2, CORO1A, DUSP11, EEF2, EIF1B, EIF4A2, HSPB1, HSPE1, HNRNPA0, HNRNPC, KCTD12, PDIA3, RPL10A, RPL11, RPL18A, RPL24, RPL26, RPL28, RPL29, RPL30, RPL32, RPL7, RPL7A, RPS11, RPS12, RPS18, RPS20, RPS23, RPS24, RPS25, RPS27, RPS27A, RPS7, RPS9, SRSF3, YWHAG |
| GO:0003723~RNA binding | 12 | FUS, DUSP11, NCL, RPL10A, RPL30, RPL34, RPL37, RPL38, RPL7A, RPS15, SRSF3, ZMAT2 |
| GO:0000166~nucleotide binding | 9 | FUS, RBM8A, U2AF1, HNRNPA0, HNRNPA2B1, HNRNPC, NCL, RPS24, SRSF3 |
| GO:0003729~mRNA binding | 8 | RBM8A, C1QBP, HNRNPC, MRPL13, RPL13A, RPL7, RPS3, RPS5 |
| GO:0019843~rRNA binding | 6 | RPL11, RPL37, RPS11, RPS18, RPS5, RPS9 |
| GO:0098641~cadherin binding involved in cell-cell adhesion | 4 | EEF2, RPL24, RPL29, RPL7A |
| GO:0042288~MHC class I protein binding | 2 | ATP5F1B, TUBB |
| GO:0003785~actin monomer binding | 2 | CORO1A, TMSB10 |
| GO:0008199~ferric iron binding | 2 | ACP5, FTH1 |
| **Kegg Pathway** | | |
| bta03010:Ribosome | 42 | ENSBTAG00000040435, MRPL13, RPL10, RPL10A, RPL11, RPL13A, RPL18A, RPL21, RPL24, RPL26, RPL27, RPL27A, RPL28, RPL29, RPL30, RPL31, RPL32, RPL34, RPL35A, RPL37, RPL38, RPL5, RPL7, RPL7A, RPS11, RPS12, RPS14, RPS15, RPS17, RPS18, RPS20, RPS21, RPS23, RPS24, RPS25, RPS27, RPS27A, RPS3, RPS5, RPS7, RPS9, RPSA |
| bta03040:Spliceosome | 6 | RBM8A, SYF2, U2AF1, HNRNPC, SRSF3, ZMAT2 |
| bta04612:Antigen processing and presentation | 5 | JSP.1, B2M, BOLA-DQA2, PSME1, PDIA3 |
| bta04145:Phagosome | 5 | JSP.1, CORO1A, BOLA-DQA2, RAC1, TUBB |
